# Resource use and economic burden of respiratory syncytial virus among older adults in real-world clinical practice across five European countries

**DOI:** 10.3389/fpubh.2026.1773556

**Published:** 2026-03-19

**Authors:** Diana Mendes, James Lucas, Emily Quinones, Katherine Li, Stephane Fievez, Caroline Lade, Roberto Di Virgilio, Alejandra López-Ibáñez de Aldecoa, James Piercy, Tim Holbrook, Reiko Sato

**Affiliations:** 1 Pfizer Ltd., Tadworth, United Kingdom; 2 Adelphi Real World, Bollington, United Kingdom; 3 Pfizer France, Paris, France; 4 Pfizer Pharma GmbH, Berlin, Germany; 5 Pfizer Italia, Rome, Italy; 6 Pfizer S.L.U, Alcobendas, Madrid, Spain; 7 Pfizer Inc, Collegeville, PA, United States

**Keywords:** economic burden, European, healthcare resource utilization, real-world evidence, respiratory syncytial virus

## Abstract

**Introduction:**

The impact of respiratory syncytial virus (RSV) is increasingly being recognized in older adults, however economic burden data is sparse. This study describes the burden of RSV and associated direct and indirect costs in adults aged 60 + years in real-world clinical practice across five European countries.

**Methods:**

Data were drawn from the Adelphi RSV Disease Specific Program (DSP™), a cross-sectional survey of physicians and patients in France, Germany, Italy, Spain, and the United Kingdom conducted December 2023–June 2024. Patients aged 60 + with a confirmed RSV diagnosis were included. Physicians provided data on resource use and supportive care needs, and patients on paid and unpaid productivity loss. Country-specific unit costs were used to calculate direct and indirect costs. Patients were stratified by symptom duration from onset (acute- [A-]RSV: <4, ongoing- [O-]RSV: 4– < 12, and post-acute- [P-]RSV: 12–52 weeks) and need for hospitalization (hospitalized [H-]RSV). All analyses were descriptive.

**Results:**

Physicians (*n* = 682) provided data on 1,581 patients with RSV (15.0% A-RSV, 12.4% O-RSV, 37.2% P-RSV, and 35.4% H-RSV). For their current RSV infection, patients had a mean of 4.3 visits to any healthcare provider (A-RSV: 3.1–H-RSV: 4.6). H-RSV patients received nearly four times as many tests as A-RSV patients, with 54.8% receiving drugs (versus <37% in other subgroups). Overall, 87.4% of H-RSV patients stayed in hospital overnight (mean stay duration: 7.2 nights). Of all patients, 36.8% required supportive care, with 93.3–98.4% receiving non-professional care. Mean overall work impairment was 53.0% (*n* = 24), with 18.0% of the 373 patients who responded reporting unpaid productivity loss. Mean direct costs per patient ranged from €320–€6,900 for A-RSV and H-RSV respectively, with hospitalization driving costs in H-RSV. Drug costs trended higher with disease duration. The indirect cost of supportive care was consistent across groups (€140–€191), meanwhile paid productive loss cost was between €52–€797 and unpaid productivity loss between €25–€415 in A-RSV and P-RSV, respectively.

**Conclusion:**

RSV poses a high economic burden in older adults across disease stages, increasing numerically with symptom duration and need for hospitalization, highlighting a need for prevention and continued monitoring.

## 1 Introduction

Respiratory Syncytial Virus (RSV) is a common cause of acute respiratory illness and is characterized by nasal congestion, fever, cough, and fatigue (1). Though RSV is well recognized as a cause of acute respiratory infections in young children, its impact is increasingly being recognized in older adults and in those with underlying health conditions (2). It is responsible for an estimated 5.1% of all respiratory infections in older adults in Europe annually, peaking at 6.7% during seasonal peaks, and proving fatal in 8.2% of medically attended cases (i.e., those that saw a medical professional for their respiratory infection) (3).

Although generally mild, RSV can cause severe lower respiratory tract symptoms, including shortness of breath and wheezing, and can lead to more serious complications such as bronchitis and pneumonia, particularly in older adults (over 60 years) and those with underlying conditions (1, 3). While age itself is a risk factor for severe RSV, other risk factors include chronic cardiac disease, chronic respiratory disease, diabetes mellitus, chronic liver disease, chronic kidney disease, immunosuppression, and neurological disorders (4). Given that comorbidities increase with advanced age, older adults in particular are at higher risk for severe RSV. Serious complications of RSV can have lasting effects on the cardiovascular and pulmonary system (5–9).

The economic impact of RSV has been well studied in infants, however, there is limited data on resource utilization and economic burden associated with RSV infection in older adults (3, 10–13). In a recent meta-analysis in adults over 18 years old, the highest rate of RSV-related hospitalizations and outpatient visits was consistently reported to be among adults aged ≥65 years (14). Recent studies found that more than two thirds of hospitalization costs due to RSV in Germany and Spain occurred in those aged 60+ (4, 15). In Germany, it was also found that costs were much higher in those with severe RSV (4). The recent introduction of RSV vaccines (16–18) provides an opportunity for public health interventions to prevent RSV associated illness. It is therefore important to fully characterize the economic burden associated with RSV infection, to identify key areas and populations where prevention is most needed. This study aims to fill this knowledge gap by describing resource utilization, supportive care needs, societal burden and direct and indirect costs associated with RSV in adults aged 60 + years presenting in real-world clinical practice across five countries in Europe.

## 2 Methods

Data were drawn from the Adelphi Real World RSV Disease Specific Program (DSP™), a cross-sectional survey with elements of retrospective data collection from physicians and their consulting patients. This survey was conducted between December 2023 and June 2024 in France, Germany, Italy, Spain, and the United Kingdom. This period was intended to align with the RSV season. DSPs are large, independent, multinational, impartial data sources not designed to address any pre-specified research questions or hypotheses. The general DSP methodology has previously been described (19, 20), validated (21), and shown to be consistent over time (22).

A sample of physicians was recruited by local fieldwork agents following a short screening questionnaire. Physician types included non-hospital based primary care physicians (PCPs) or geriatricians, and hospital-based pulmonologists or infectious disease specialists. These physicians provided data for their next four consecutively consulting patients with RSV. To be included in this analysis, patients had to be aged 60 + years, have a RSV diagnosis confirmed by molecular testing, have been experiencing symptoms at the time of the survey for less than 1 year, have a known symptom onset date, and must not have been residing in a long-term care facility.

Physicians were asked to complete patient record forms containing questions on patient demographics, clinical characteristics, resource utilization, treatment, and supportive care needs. Symptoms and risk factors were selected from a predefined list of concomitant conditions associated with severe RSV (4, 23, 24). Time since symptom onset was defined as the time from when the patient first experienced a symptom associated with their current RSV infection to survey. Time from diagnosis was defined as the time in months from when RSV was first confirmed via molecular testing.

These same patients were asked to complete a patient self-complete form on employment status and hours of work missed in the past 7 days. This form also contained the work productivity and activity impairment questionnaire (WPAI), a validated patient reported outcome measure from which percent work time missed due to illness (absenteeism), percentage impairment while working (presenteeism), percentage overall work impairment, and percentage overall activity impairment were calculated (25). For the purposes of this study the methodology set out to capture sufficient numbers of acute, persistent and hospitalized patients to adequately assess resource use and costs, meaning sample sizes are not intended to reflect the true epidemiology of RSV in terms of rate of hospitalization.

### 2.1 Analysis

To evaluate how RSV burden differs by time since symptom onset and need for hospitalization, patients were categorized into four groups. Patients consulting a PCP at survey with no history of hospitalization due to RSV infection were classified as acute RSV (A-RSV; symptom duration <4 weeks), ongoing RSV (O-RSV; 4– < 12 weeks), or post-acute RSV (P-RSV; 12–52 weeks). Patients with a history of hospitalization for their current RSV infection were classified as hospitalized RSV (H-RSV) regardless of time since symptom onset (0–52 weeks) or whether they were consulting in a primary or secondary care environment. Results were also stratified for supplementary analysis by age, presence/absence of risk factors for severe RSV, and country. Risk factors for severe RSV only included those comorbidities that were diagnosed prior to RSV onset, where this information was known.

Physicians reported whether a patient had visited a PCP, infectious disease specialist, pulmonologist, emergency physician, or geriatrician, as well as the number of visits to each physician type. Patients who saw a physician type but for whom number of visits was not recorded, were counted as visiting that physician type only once, hence potentially underestimating the number of HCP visits reported. The total number of supportive care hours received by RSV patients was split by professional (paid) and non-professional (informal). Total number of caregiver hours per week was capped at 168 h as this represented 24-h 7 days a week care.

Direct and indirect costs associated with RSV were calculated on a country-specific basis using unit costs sourced from national healthcare systems and regulatory bodies. All unit costs published prior to 2024 were adjusted for inflation and are presented in Euros (€). Unit costs can be found in Supplementary Table S1. Direct costs included cost of HCP visits, testing, drugs, hospitalization, and procedures received in hospital. Indirect costs included the cost of receiving supportive care and those associated with paid and unpaid productivity loss. While some formal supportive care costs may have been funded directly by the public sector, we have conservatively assumed all supportive care was supported indirectly by the patient. Costs of HCP visits and testing were calculated based on mean number of HCP visits or tests per patient multiplied by the unit cost associated with that resource. In patients where the number of HCP visits was unknown (<12% of patients), mean costs were imputed. The cost of drugs was calculated using the mean duration patients were prescribed each medication. Hospitalization costs were calculated on a per night basis, with the unit costs associated with a night in hospital multiplied by the mean number of overnight stays (length of stay). If the patient was discharged on the same day as admission (without overnight stay), a flat rate was applied for one admission to the hospital. Costs of supportive care were calculated by multiplying the number of supportive care hours patients received per week by unit costs for an hour of supportive care and the duration of disease. The cost of paid productivity loss was calculated for all those that completed the relevant sections of the patient self-complete form, for those who were retired costs associated with paid work were assumed to be €0. For calculating the cost of paid productivity loss, the mean overall percentage work impairment as measured by the WPAI was multiplied against the hourly wage in each country and, assuming a 40-h working week, by the average disease duration for each group. Unpaid productivity loss was defined as the number of hours patients missed providing unpaid care to others or conducting voluntary work. Patients specified how many hours they spent caring for others or working voluntarily and how often they had to miss this due to RSV; this was multiplied against the hourly wage and disease duration for each group.

All data analysis was descriptive. Continuous variables were presented as mean (standard deviation; SD) or median (interquartile range; IQR) as appropriate and categorical variables as % (n). All data were analyzed using survey reporter (IBM Corp. Released 2023. IBM SPSS Statistics for Windows, Version 29.0.2.0 Armonk, NY: IBM Corp).

### 2.2 Ethics statement

The DSP was conducted in accordance with European Pharmaceutical Market Research Association guidelines as well as all relevant national guidelines at time of survey including the Declaration of Helsinki (26). The RSV DSP protocol was submitted to the Pearl institutional review board (IRB number: 2023–0459), where it was deemed exempt from ethical approval. No personally identifiable data was collected as part of the DSP. All data was pseudonymized by assigning a code at data collection to allow for matching between patient and physicians. All participants provided informed consent prior to data collection.

## 3 Results

### 3.1 Demographics and clinical characteristics

Overall, 682 physicians provided data on 1,581 patients with RSV (France: *n* = 213, Germany: *n* = 438, Italy: *n* = 403, Spain: *n* = 371, UK: *n* = 156). Physician demographics can be found in Supplementary Table S2. Of the 1,581 patients, 15.0% (*n* = 237) had A-RSV, 12.4% (*n* = 196) O-RSV, 37.2% (*n* = 588) P-RSV, and 35.4% (*n* = 560) H-RSV. Median age was numerically similar across the four cohorts (range 69 to 71 years) and the median (IQR) time since RSV symptom onset was 79.0 (16.5, 131.0) days. The proportion of patients with risk factors for severe RSV ranged from 62.0% in A-RSV to 85.9% in H-RSV. Most patients were retired (70.1%). For further demographics see Table 1.

**Table 1:** Patient demographics and clinical characteristics.

Patient demographics	Overall (*n* = 1,581)	A-RSV (*n* = 237)	O-RSV (*n* = 196)	P-RSV (*n* = 588)	H-RSV (*n* = 560)
Country					
France	213 (13.5%)	25 (10.5%)	31 (15.8%)	83 (14.1%)	74 (13.2%)
Germany	438 (27.7%)	92 (38.8%)	45 (23.0%)	142 (24.1%)	159 (28.4%)
Italy	403 (25.5%)	52 (21.9%)	70 (35.7%)	179 (30.4%)	102 (18.2%)
Spain	371 (23.5%)	54 (22.8%)	36 (18.4%)	134 (22.8%)	147 (26.3%)
United Kingdom	156 (9.9%)	14 (5.9%)	14 (7.1%)	50 (8.5%)	78 (13.9%)
Age, median (IQR) years	70.0 (66.0, 76.0)	69.0 (65.5, 72.5)	70.0 (65.0, 75.0)	70.0 (66.0, 76.0)	71.0 (66.0, 78.0)
Male, n (%)	788 (49.8%)	120 (50.6%)	96 (49.0%)	286 (48.6%)	286 (51.1%)
Body mass index, median (IQR)	25.7 (23.5, 28.4)	25.3 (23.5, 27.7)	25.9 (23.7, 28.1)	25.7 (23.5, 28.2)	26.1 (23.4, 29.3)
Smoking status, n (%)					
Current/ Ex-smoker	926 (59.9%)	104 (45.4%)	114 (59.1%)	332 (57.5%)	376 (68.6%)
Never smoked	621 (40.1%)	125 (54.6%)	79 (40.9%)	245 (42.5%)	172 (31.4%)
Do not know	34 (2.2%)	8 (3.4%)	3 (1.5%)	11 (1.9%)	12 (2.1%)
Ethnicity, n (%)^1^	*n* = 1,368	*n* = 212	*n* = 165	*n* = 505	*n* = 486
White	1,311 (95.8%)	205 (96.7)	154 (93.3%)	482 (95.4%)	470 (96.7%)
Other^2^	60 (4.4%)	7 (3.3%)	11 (6.7%)	25 (5.0%)	17 (3.5%)
Time since RSV symptom onset, median (IQR) days	79.0 (16.5, 131.0)	12.0 (6.0, 19.0)	49.0 (35.3, 63.0)	132.0 (109.0, 173.0)	55.0 (21.0, 104.8)
Time since diagnosis of RSV, median (IQR) days	79.0 (26.5, 131.0)	8.0 (3.0, 15.5)	41.0 (30.0, 60.0)	124.0 (101.0, 163.0)	48.0 (16.0, 93.5)
Concomitant conditions, mean (SD) number	1.7 (1.4)	1.2 (1.1)	1.6 (1.4)	1.6 (1.3)	2.0 (1.4)
Patients with concomitant conditions, n (%)	*n* = 1,581	*n* = 237	*n* = 196	*n* = 588	*n* = 560
0 concomitant condition	255 (16.2%)	60 (25.4%)	45 (23.0%)	99 (16.9%)	255 (16.2%)
1 concomitant condition	547 (34.6%)	104 (43.9%)	57 (29.1%)	200 (34%)	186 (33.2%)
2 concomitant conditions	356 (22.5%)	39 (16.5%)	44 (22.4%)	144 (24.5%)	129 (23.0%)
3 + concomitant conditions	772 (48.9%)	69 (29.2%)	90 (45.9%)	285 (48.6%)	328 (58.7%)
Patients with risk factors for severe RSV, n (%)	1,177 (74.4%)	147 (62.0%)	131 (66.8%)	418 (71.1%)	481 (85.9%)
Employment status, n (%)	*n* = 1,571	*n* = 235	*n* = 196	*n* = 587	*n* = 553
Retired	1,108 (70.5%)	167 (71.1%)	129 (65.8%)	397 (67.6%)	415 (75.0%)
Working full/part time	234 (14.9%)	36 (15.3%)	36 (18.4%)	89 (15.2%)	73 (13.2%)
Other^3^	229 (14.6%)	32 (13.6%)	31 (15.8%)	101 (17.2%)	65 (11.8%)
Insurance type, n (%)	*n* = 1,571	*n* = 237	*n* = 193	*n* = 586	*n* = 555
Public	1,302 (82.9%)	200 (84.4%)	154 (79.8%)	468 (79.9%)	480 (86.5%)
Public and private, insurance covered	206 (13.1%)	27 (11.4%)	32 (16.6%)	85 (14.5%)	62 (11.2%)
Other	63 (4.0%)	10 (4.2%)	7 (3.6%)	33 (5.6%)	13 (2.3%)

Answers of “Do not know” have been excluded from resource calculations. Across the total population, no more than 11.4% of physicians provided this answer for any one variable. RSV, acute respiratory syncytial virus; A-RSV, acute RSV; O-RSV, ongoing RSV; P-RSV, post-acute RSV; H-RSV, hospitalized RSV; IQR, interquartile range.

1 Ethnicity data not captured in France.

2 Other ethnicities includes: Black African or Caribbean, American Indian, Indigenous American, or Alaska Native, East or Southeast Asian, South Asian (Indian subcontinent), Middle Eastern or North African, and Other (specify).

3 Other employment status includes: Homemaker, on long term sick leave, and unemployed.

### 3.2 Resource utilization, supportive care needs, and productivity loss

Table 2 shows the burden of disease for each subgroup. For their most recent RSV episode, patients had a mean (SD) of 4.3 (3.3) visits to any HCP, ranging from 3.1 (2.6) in A-RSV to 4.6 (3.8) in H-RSV. Most patients without RSV hospitalization had at least one PCP visit in the last 12 months (Table 2), with between 2.4 (2.6) consultations in A-RSV and 3.7 (3.3) in P-RSV. Half (53.6%) of H-RSV patients consulted a PCP for their RSV, with an average of 1.5 (2.8) visits per H-RSV patient. Meanwhile, 42.2–49.8% of patients without history of RSV hospitalization and 95.9% of H-RSV patients consulted a specialist.

**Table 2:** Resource utilization, supportive care needs, and productivity loss.

	Overall (*n* = 1,581)	A-RSV (*n* = 237)	O-RSV (*n* = 196)	P-RSV (*n* = 588)	H-RSV (*n* = 560)
Physician type visted					
Visited a PCP, n (%)	1,144 (72.4%)	187 (78.9%)	159 (81.1%)	498 (84.7%)	300 (53.6%)
Visited a specialist*, n (%)	1,027 (65%)	100 (42.2%)	97 (49.5%)	293 (49.8%)	537 (95.9%)
Number of HCP visits per patient, mean (SD)	4.3 (3.3)	3.1 (2.6)	3.9 (2.8)	4.6 (3.1)	4.6 (3.8)
PCP	2.6 (3.1)	2.4 (2.6)	2.9 (2.9)	3.7 (3.3)	1.5 (2.8)
Infectious disease specialist	0.7 (1.9)	0.3 (1.0)	0.5 (1.2)	0.4 (1.2)	1.1 (2.7)
Pulmonologist	0.9 (1.7)	0.3 (0.8)	0.5 (1.0)	0.6 (1.2)	1.6 (2.3)
Emergency physician	0.2 (0.5)	0.1 (0.2)	0.1 (0.3)	0.1 (0.4)	0.5 (0.7)
Geriatrician	0.1 (0.7)	0.0 (0.4)	0.0 (0.3)	0.1 (0.4)	0.2 (1.0)
Number of tests (to diagnose or monitor) per patient, mean (SD)	6.2 (8.3)	2.5 (1.9)	3.7 (3.7)	5.0 (5.9)	9.7 (11.3)
RT-PCR	1.1 (1.0)	0.81 (0.8)	0.9 (0.9)	1.1 (1.2)	1.3 (0.9)
Rapid antigen	0.9 (1.3)	0.6 (0.9)	0.89 (1.3)	1.0 (1.4)	0.8 (1.3)
Pulse oximetry	1.1 (2.1)	0.5 (1.2)	0.8 (1.6)	1.1 (2.2)	1.4 (2.5)
Chest x-ray	1.0 (1.1)	0.4 (0.6)	0.5 (0.7)	0.8 (0.9)	1.6 (1.2)
Received medication (currently or previously), n (%)	659 (42.2%)	74 (31.2%)	71 (36.6%)	211 (36.6%)	303 (54.8%)
Most recent medication prescribed, n (%)	*n* = 659	*n* = 74	*n* = 71	*n* = 211	*n* = 303
Ribavirin	417 (63.3%)	42 (56.8%)	45 (63.4%)	122 (57.8%)	208 (68.6%)
Antibiotic	44 (6.7%)	4 (5.4%)	3 (4.2%)	4 (1.9%)	33 (10.9%)
Corticosteroid	14 (2.1%)	1 (1.4%)	2 (2.8%)	0 (0.0%)	11 (3.6%)
Analgesic	11 (1.7%)	3 (4.1%)	1 (1.4%)	6 (2.8%)	1 (0.3%)
Other	178 (27.0%)	25 (33.8%)	25 (33.8%)	79 (37.4%)	54 (17.8%)
Over-the-counter medication taken, n (%)	*n* = 1,278	*n* = 209	*n* = 164	*n* = 463	*n* = 442
Paracetamol	811 (63.5%)	106 (50.7%)	102 (62.2%)	283 (61.1%)	320 (72.4%)
Cough suppressants	521 (40.8%)	106 (50.7%)	61 (37.2%)	193 (41.7%)	161 (36.4%)
Ibuprofen	507 (39.7%)	108 (51.7%)	65 (39.6%)	204 (44.1%)	130 (29.4%)
Nasal decongestants	389 (30.4%)	89 (42.6%)	43 (26.2%)	142 (30.7%)	115 (26.0%)
Other	102 (8.0%)	9 (4.3%)	12 (7.3%)	26 (5.6%)	55 (12.4%)
Received supportive care (currently), n (%)	569 (36.8%)	75 (31.9%)	63 (33.7%)	191 (33.2%)	240 (43.7%)
Supportive care type, n (%)	*n* = 569	*n* = 75	*n* = 63	*n* = 191	*n* = 240
Non-professional**	539 (94.7%)	70 (93.3%)	62 (98.4%)	179 (93.7%)	228 (95.0%)
Professional	58 (10.2%)	5 (6.7%)	7 (11.1%)	20 (10.5%)	26 (10.8%)
Total number of care hours received per week per patient, mean (SD)	*n* = 294	*n* = 44^†^	*n* = 39^†^	*n* = 104	*n* = 107
Non-professional	29.9 (33.6)	27.7 (30.4)	23.2 (24.7)	30.13 (37.2)	33.0 (34.0)
	*n* = 42	*n* = 3^†^	*n* = 5^†^	*n* = 15^†^	*n* = 19^†^
Professional	37.2 (42.1)	30.7 (16.2)	20.6 (14.7)	47.3 (50.2)	34.5 (42.7)
Hospitalization	*n* = 560	*n* = 0	*n* = 0	*n* = 0	*n* = 560
Discharged on same day as admission, n (%)	35 (7.3%)	-	-	-	35 (7.3%)
Requiring one or more overnight stays, n (%)	417 (87.4%)	-	-	-	417 (87.4%)
Total number of nights spent in hospital, mean (SD)	7.2 (5.5)	-	-	-	7.2 (5.5)
Total number of nights spent in hospital (patients never admitted to ICU), mean (SD)	*n* = 349	*n* = 0	*n* = 0	*n* = 0	*n* = 349
	6.6 (5.1)	-	-	-	6.6 (5.1)
Total number of nights spent in hospital (patients admitted to ICU), mean (SD)	*n* = 67	*n* = 0	*n* = 0	*n* = 0	*n* = 67
	10.1 (5.9)	-	-	-	10.1 (5.9)
Admitted to ICU, n (%)	69 (14.5%)	-	-	-	69 (14.5%)
Nights spent in ICU, mean (SD) (of those admitted to ICU)	3.3 (2.0)	-	-	-	3.3 (2.0)
Received procedures during hospitalization for most recent RSV infection, n (%)	415 (75.5%)	-	-	-	415 (75.5%)
Procedures received for most recent infection during hospitalization, n (%)	*n* = 415	*n* = 0	*n* = 0	*n* = 0	*n* = 415
Receiving oxygen/IV fluids	399 (96.1%)	-	-	-	399 (96.1%)
Nasal bulb suctioning	54 (13.0%)	-	-	-	54 (13.0%)
Mechanical ventilation	44 (10.6%)	-	-	-	44 (10.6%)
Receiving feeding tubes	20 (4.8%)	-	-	-	20 (4.8%)
Intubation	13 (3.1%)	-	-	-	13 (3.1%)
WPAI: percentage overall work impairment, mean (SD)	*n* = 29^†^	*n* = 8^†^	*n* = 6^†^	*n* = 10^†^	*n* = 5^†^
	53.0% (27.1)	48.2% (25.3)	52.1% (30.7)	57.2% (30.5)	53.3% (25.9)
WPAI: percentage overall activity impairment, mean (SD)	*n* = 405	*n* = 110	*n* = 43^†^	*n* = 123	*n* = 129
	51.3% (24.7)	50.4% (22.5)	47.4% (26.7)	46.5% (24.4)	57.9% (24.8)
Missed work due to RSV in past 7 days, n (%)	*n* = 51	*n* = 12^†^	*n* = 7^†^	*n* = 13^†^	*n* = 19^†^
	37 (72.5%)	8 (66.7%)	6 (85.7%)	8 (61.5%)	15 (78.9%)
Missed hours of paid work due to RSV in the past 7 days, mean (SD)	19.0 (17.7)	15.3 (15.8)	15.7 (10.9)	10.6 (14.2)	28.2 (19.6)
Missed unpaid activity due to RSV, n (%)	*n* = 373	*n* = 101	*n* = 40	*n* = 114	*n* = 118
	67 (18.0%)	14 (13.9%)	6 (15.0%)	14 (12.3%)	33 (28.0%)
Hours of unpaid activity lost per week, mean (SD)	*n* = 67^†^	*n* = 14^†^	*n* = 6^†^	*n* = 14^†^	*n* = 33^†^
	6.7 (7.4)	5.1 (3.8)	8.0 (8.7)	8.7 (9.7)	6.3 (7.0)
Supporting or caring for a family member	*n* = 19^†^	*n* = 5^†^	*n* = 2^†^	*n* = 6^†^	*n* = 6^†^
	9.5 (8.9)	5.6 (2.7)	1.0 (1.4)	15.2 (12.5)	10.0 (6.4)
Supporting or caring for a friend	*n* = 2^†^	*n* = 0	*n* = 2^†^	*n* = 0	*n* = 0
	4.0 (5.7)	-	4.0 (5.7)	-	-
Assisting in household activities	*n* = 31^†^	*n* = 6^†^	*n* = 2^†^	*n* = 6^†^	*n* = 17^†^
	6.9 (5.6)	5.7 (2.7)	12.0 (17.0)	3.7 (2.3)	7.9 (5.3)
Voluntary work	*n* = 7^†^	*n* = 2^†^	*n* = 1^†^	*n* = 1^†^	*n* = 3^†^
	3.1 (2.0)	5.0 (1.4)	0.0 (−)	2.0 (−)	3.3 (1.2)
Other	*n* = 4^†^	*n* = 0	*n* = 2^†^	*n* = 1^†^	*n* = 1^†^
	6.0 (6.1)	-	7.0 (9.9)	7.0 (−)	3.0 (−)

For HCP visits answers of “Do not know” were imputed as the mean value for other patients. PCP, primary care physician; RF, risk factors; HCP, health care provider; SD, standard deviation; RT-PCR, reverse-transcription polymerase chain reaction; WPAI, work productivity and impairment scale. †Caution low base size.

*Specialist includes infectious disease specialist, pulmonologist, emergency physician, geriatrician.

**Non-professional caregiver includes partner/spouse, parent/guardian, child under 18 years, child over 18 years, other relative(s), friend(s)/neighbor(s), other non-professional caregiver(s).

The mean (SD) number of tests used to diagnose and monitor RSV ranged from 2.5 (1.9) in A-RSV to 9.7 (11.3) in H-RSV, with H-RSV patients receiving nearly four times as many tests as A-RSV patients (Table 2). Consultation and testing rates were particularly high among those aged 75 + years with risk factors for all four RSV patient subgroups (Supplementary Tables S3–S6). Approximately a third of A-RSV, O-RSV, and P-RSV patients received medication for their RSV, most commonly ribavirin (Table 2). In the H-RSV group, 54.8% of patients received medication for their RSV, with 68.6% of those receiving ribavirin and 10.9% receiving antibiotics (compared to <5.4% of patients in other groups; Table 2).

Most H-RSV patients had an overnight hospital stay (87.4%) with a mean (SD) duration of 7.2 (5.5) nights per hospitalization. Of those, 14.5% had an ICU admission, spending a mean of 3.3 (2.0) nights in ICU and a mean of 10.1 (5.9) nights in a general ward. Excluding those admitted to ICU, the mean (SD) duration of hospital stay was 6.6 (5.1) nights. Most H-RSV patients had oxygen/intravenous (IV) fluids administered (96.1%) and 10.6% had mechanical ventilation (Table 2).

Overall, 36.8% of patients required supportive care, with 6.7–11.1% needing a professional caregiver and 93.3–98.4% needing a non-professional caregiver across the four RSV subgroups (Table 2). The need for a professional caregiver was mainly reported in those aged 75 + years with risk factors (Supplementary Tables S3–S6).

**Figure 1:**
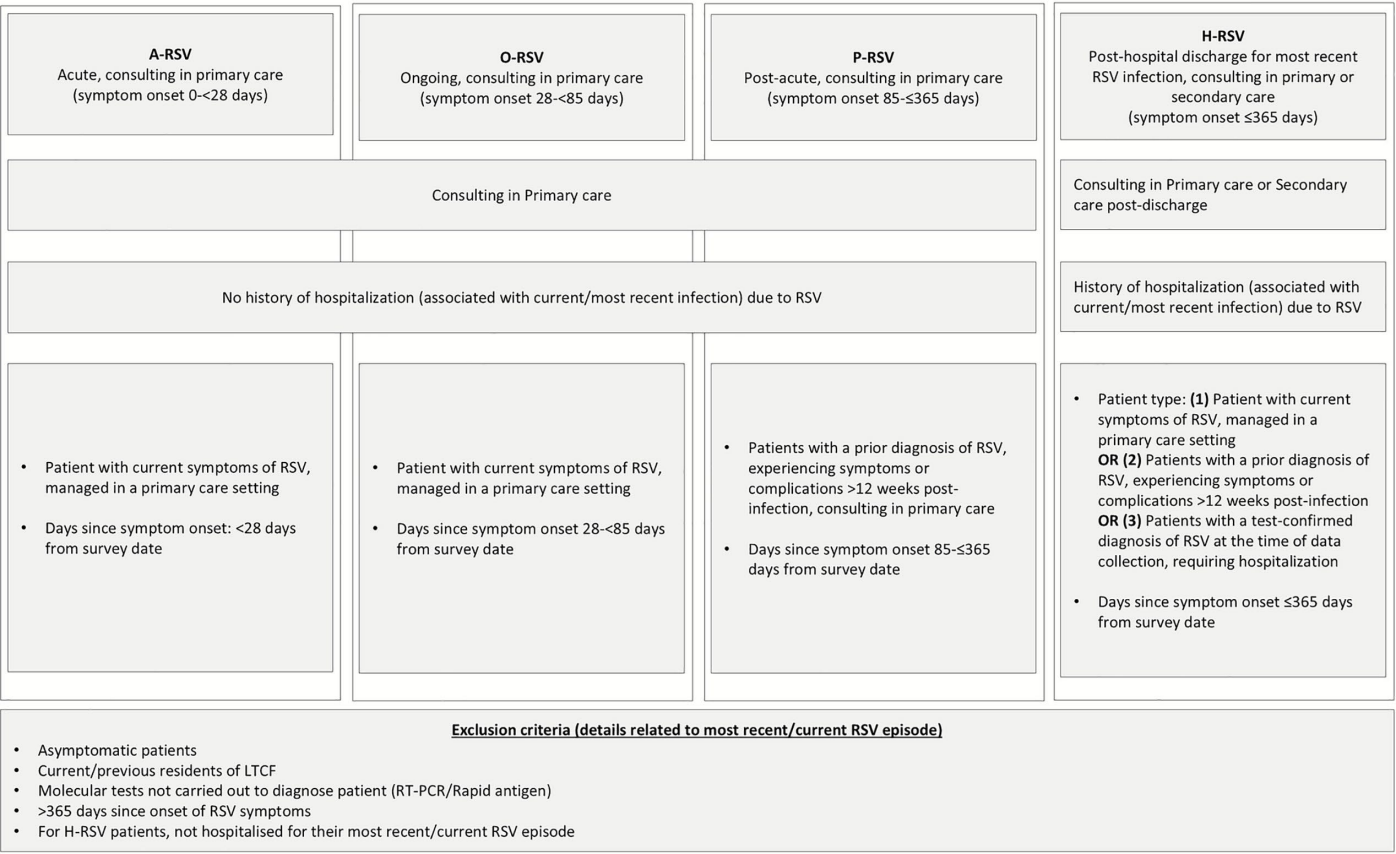
Subgroup definitions with inclusion and exclusion criteria for each subgroup. RSV, respiratory syncytial virus; LTCF, long-term care facility; RT-PCR, reverse transcription polymerase chain reaction.

Among 57 patients aged 60–74 who completed the patient self-complete form and reported working, 72.5% reported missing work due to RSV in the week prior to survey (ranging from 61.5 to 85.7%; Table 2). H-RSV patients missed a mean (SD) of 28.2 (19.6) hours work during the 7 days prior to survey compared to 5.3 (15.8) hours in A-RSV (Table 2; Figure 1). Patients reported a mean (SD) overall percentage work impairment of 53.0% (27.1%), ranging from 48.2% (25.3%) in A-RSV to 57.2% (30.5%) in P-RSV. Overall activity impairment was also high, with patients experiencing activity impairment due to their RSV in the 7 days prior to survey around half of the time. Meanwhile, 18.0% reported unpaid productivity loss, missing a mean (SD) of 6.7 (7.4) hours in the previous week. Country-specific resource use can be found in Supplementary Table S7.

### 3.3 Costs

**Figure 2:**
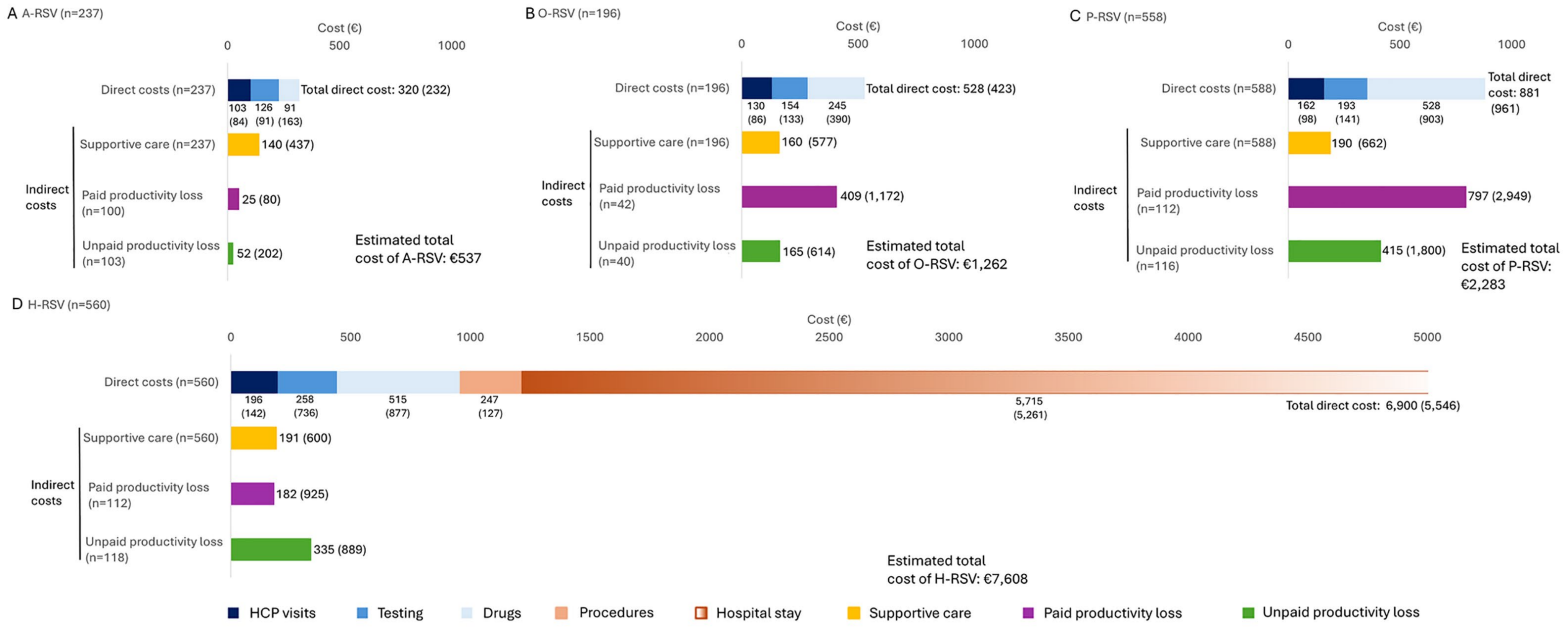
RSV-related cost per patient and subgroup, mean (SD), Euros (€). A-RSV, acute respiratory syncytial virus; O-RSV, ongoing RSV; P-RSV, post-acute RSV; H-RSV, hospitalized RSV.

All direct costs trended higher with disease duration and need for hospitalization (Figure 2). The mean (SD) direct cost to healthcare per patient ranged from €320 (€232) for A-RSV to €6,900 (€5,546) for H-RSV. In A-RSV, direct costs were contributed to equally by HCP visits (€103, 22.7% of total direct costs), testing (€126, 27.4%), and drugs (€91, 19.4%). Drug costs trended higher with disease duration across non-hospitalized patient subgroups, with medication use accounting for nearly a third of direct costs in O-RSV and over half of direct costs in P-RSV (Figure 2). In H-RSV, costs were principally driven by the cost of the hospitalization itself at €5,714 (€5,260).

Regarding indirect cost, costs of supportive care received were relatively consistent between groups, trending slightly higher with longer disease duration and the need for hospitalization, ranging from €140 (€437) in A-RSV to €191 (€570) in H-RSV. In all, the costs of paid and unpaid productivity loss were calculable for 100 and 103 A-RSV patients, 42 and 40 O-RSV patients, 112 and 116 P-RSV patients, and 112 and 118 H-RSV patients, respectively. Paid productivity loss had an indirect cost of between €52 (€102) and €797 (€2,949) and unpaid productivity loss of €25 (€80) and €415 (€1,800) in A-RSV and P-RSV, respectively. The total estimated cost of RSV per patient was €537 for A-RSV, €1,262 for O-RSV, €2,283 for P-RSV, and €7,639 for H-RSV, though the number of patients contributing data on productivity loss was smaller than other costs. The economic burden among P-RSV and H-RSV patients was numerically higher for those with risk factors for severe RSV, with overall direct costs of €933 (€958) and €7,167 (€5,678), respectively, compared to €753 (€958) and €5,276 (€4,354) in those without risk factors (Supplementary Table S8). Country-specific RSV-related costs can be found in Supplementary Table S9.

## 4 Discussion

In this study, we assessed the real-world direct and indirect economic burden associated with RSV in adults aged 60 years and over across all stages of illness. Resource use and the direct costs associated with HCP visits, testing, and drug use were numerically higher with increasing disease duration, with advanced age, and presence of risk factors adding to the burden. This burden was most pronounced among those with P-RSV and H-RSV, with the costs of hospitalization and procedures adding greatly to the economic burden of H-RSV. In addition, RSV had a substantial indirect economic burden in the form of societal costs associated with the need for supportive care and loss of productivity among patients even beyond the acute phase of illness.

Costs of HCP visits and testing represented a substantial proportion of direct costs in patients with A-RSV, with comparable costs seen across O-RSV, and P-RSV. The costs of HCP visits were numerically highest in H-RSV, with H-RSV patients having a descriptively similar number of consultations to those with P-RSV despite time from diagnosis in H-RSV being half that of O-RSV. The additional cost of consultations in H-RSV is likely driven by increased need for, and access to, specialist care compared to P-RSV. H-RSV patients also had nearly twice as many tests as P-RSV patients despite shorter disease time course, which likely reflects the relative availability of tests to monitor RSV in an inpatient setting, as well as the increased disease severity associated with need for hospitalization. Overall, this demonstrates the high burden of consultations and testing experienced by H-RSV patients in a relatively short period of time.

The use of ribavirin, although not an antiviral specific to RSV, was common across all four subgroups (prescribed to over 60% of patients) despite its high cost. Interestingly, antibiotics were prescribed to a tenth of patients with H-RSV and 7% of patients overall. This could be due to the high rates of pneumonia and other potential bacterial complications observed in older adults hospitalized for RSV (27). Antibiotic use and resistance represent a significant clinical and economic burden globally suggesting that use of antibiotics for RSV could have far-reaching consequences beyond what we assess here (28–30).

Direct costs for H-RSV were 21, 13, and 8 times those reported for A-RSV, O-RSV, and P-RSV, respectively, with over 80% of these costs associated with the hospitalization itself. This aligns with previous studies in France, Germany, Italy, and Spain that showed high costs associated with inpatient stays (4, 15, 31, 32). These costs also varied by country and presence of risk factors validating our estimates (4, 15, 31, 32). Most H-RSV patients had pre-existing risk factors for severe RSV. RSV is known to trigger exacerbations of risk factors such as COPD which in turn leads to longer hospital stays (15, 33). ICU admittance and duration spent also aligned well with studies in patients hospitalized for RSV, with length of hospital stay generally reported at between 4 and 8 days (34–36). High costs associated with hospitalization highlight that preventing hospitalization in those most at risk is key to reducing economic burden associated with RSV.

A third of patients without history of RSV hospitalization reported needing supportive care, of whom 90% received supportive care from a non-professional caregiver (family and friends). Meanwhile, nearly half of H-RSV patients needed supportive care, likely due to the high proportion of pre-existing risk factors. Severe complications such as pneumonia are more common among those at high-risk of severe RSV, with hospitalized RSV patients more likely to have severe underlying conditions, such as COPD and heart failure, that potentially already needed supportive care (3, 37). Proportion of people receiving informal care can vary significantly by country based on cultural difference and generosity of formal care previsions (38). Though the costs of receiving supportive care were generally among the lowest due to care being provided by unpaid, non-professional caregivers, it represented a substantial burden in A-RSV, O-RSV, and P-RSV.

Paid productivity loss was high with almost half of those that worked missing work in the week prior to survey due to RSV. This cost was particularly high among O-RSV and P-RSV patients due to the prolonged time course of illness, with paid productivity loss almost equaling the direct costs of RSV. The burden of paid productivity loss is likely even higher in those who worked, as average costs are likely reduced here by the inclusion of patients who were retired (and who were therefore assigned a paid productivity loss of €0). Indeed, the cost of paid productivity loss was particularly high among those aged 60–75 years, more of whom were working (Supplementary Table S8). Levels of overall activity impairment reported were similar to overall work impairment in the week prior to survey (between 40 and 60%). Importantly, indirect burden extended beyond just paid work, with between €25 and €415 in lost time spent providing care or performing voluntary work. As calculations were based on the WPAI which has a recall period of 7 days prior to survey, the fact that P-RSV patients were still missing work due to RSV demonstrates the long-lasting negative impact of RSV on productivity. Work and activity impairment are also likely underestimated due to the voluntary nature of the patient self-complete form where healthier patients likely chose to complete the self-complete form.

Finally, the economic burden of RSV reported here is likely a conservative estimate as we have only included costs where we can source a unit cost. Similarly, we only included confirmed cases of RSV, meaning we likely did not capture the full spectrum of RSV which is often underdiagnosed due to under-testing and low diagnostic test sensitivity (2, 39, 40). Despite this, our study demonstrated a substantial economic burden associated with RSV regardless of illness stage. Though there is limited literature comparing costs associated with respiratory infections in Europe, studies in the US have found that the cost of hospitalization for RSV were at least similar to those reported for influenza in older adults (most recently reported as $27,757 vs. $22,164; *p* < 0.001, respectively), with RSV resulting in longer hospital stays (34, 41, 42). Given that across Europe RSV is estimated to be responsible for 145,102 hospitalizations in patients aged ≥65 years per year (43), we highlight that to reduce the economic burden of RSV there is a need for prophylactic intervention to protect those most vulnerable to severe complications of RSV and prevent the need for hospitalization. Since 2023, three new vaccines for RSV have been introduced to the market to prevent RSV infection in adults aged 60 + years (44). Early adoption and implementation of RSV vaccines in the UK and Scotland in older adults have demonstrated significant reduction in RSV associated hospitalization in just the first season (45, 46).

### 4.1 Study limitations

The DSP methodology has several limitations that should be considered when interpreting this study. The DSP is a voluntary survey of actively consulting patients; as such, the sample collected may not reflect the broader RSV population. The voluntary nature of the DSP means that data collected are for those willing to complete the survey. Similarly, physicians and patients could only provide data they had available and were willing to provide, leading to heterogeneous sample sizes for select datapoints. There is also the potential for recall bias from both physician and patient responses in this cross-sectional survey. While physicians were encouraged to use patient medical records to minimize recall bias, these records may have been incomplete. The cross-sectional nature of this survey also means we were unable to follow the trajectory of patient recovery. Cost analyses were based on published costs of resources, and as such, costs may not reflect actual acquisition costs for HCPs. Furthermore, when calculating costs for HCP visits, mean imputation was used to impute missing data, and, although less than 12% of patients had data missing, this could reduce the variability of data and could mean the cost of HCP visits are over- or underestimated. When calculating the cost of hospitalization, it is possible that unit costs for nights in hospital already include the cost of procedures meaning some procedure costs may be duplicated. Similarly, the assumption that those not admitted to hospital overnight accrued costs for one full day of hospitalization could also have led to overestimations of costs. Finally, costs of supportive care are likely underestimated because caregiver hours were capped at 168 as the maximum number of hours in a week, however patients with more than one caregiver may have received more than 168 h of care a week. Care should be taken when interpreting the costs associated with productivity loss reported here, standard deviation for these costs is high due to the low number of patients who worked in our sample. Furthermore, work productivity costs were based on a seven-day recall period which we multiplied by the duration of disease. This means that costs may be overestimated in acute patients, and underestimated in those who may have been recovering from illness (and therefore may have faced lower impairment in the week prior to survey). The WPAI, however, is a validated tool that is widely recognized and used in real-world studies.

## 5 Conclusion

RSV imposes a high economic burden in older adults across all stages of disease. Economic burden increased with symptom duration and need for hospitalization. The direct cost of HCP-visits, testing and drugs represented a substantial burden across all groups, with the costs of drugs particularly high in P-RSV and H-RSV. The cost of supportive care was consistent across all subgroups, whereas productivity loss was more costly among patients with longer disease duration, such as P-RSV patients. Advanced age and risk factors for RSV combined added to the direct costs associated with RSV. Overall, these findings highlight a potential opportunity for early intervention to prevent severe RSV and minimize the economic burden of RSV in older adults. Overall, these findings highlight a potential opportunity for vaccination or early treatment intervention to prevent severe RSV and minimize the economic burden of RSV in older adults. Further work is needed to assess how interventions may impact on the economic burden of RSV.

## Data Availability

The datasets presented in this article are not readily available because the dataset remains under the ownership of Adelphi Real World and is not available publicly. Requests to access the datasets should be directed to james.lucas@adelphigroup.com.
