# Supplementary material for: Resource use and economic burden of respiratory syncytial virus among older adults in real-world clinical practice across five European countries
Source: Front Public Health. 2026 Mar 19;14:1773556. doi: 10.3389/fpubh.2026.1773556 (PMC13044084; doi:10.3389/fpubh.2026.1773556)
Supplement: Supplementary file 1 [file Data_Sheet_1.pdf]

Supplementary Table S1: Unit costs

Description	Unit	Cost (€)	Reference
**One hour caregiver earning**	Their partner/spouse	25.55	[1-5]
	Their parent/guardian	25.55	[1-5]
	Their child over 18 years	25.55	[1-5]
	Other relative(s)	25.55	[1-5]
	Friend(s)/neighbor(s)	25.55	[1-5]
	Other non-professional caregiver(s)	25.55	[1-5]
	Professional caregiver	22.52	[1, 6-9]
**One HCP consultation**	PCP/GP	29.25	[10-14]
	Infectious disease specialist	36.27	[10-16]
	Pulmonologist	39.73	[10-16]
	Emergency physician	35.74	[10-13, 17-19]
	Geriatrician	35.20	[10, 12-16, 21]
**Treatment (price per-packet)**	Ribavirin	408.21	[22-25]
	Dexamethasone (Corticosteroid)	4.24	[26, 27-30]
	Levofloxacin (antibiotic)	5.26	[27, 28, 31-33]
	Diclofenac (analgesic)	0.49	[27, 28, 34-36]
**OTC medication (price per-packet)**	Ibuprofen	2.74	[37-40]
	Paracetamol	3.19	[39-43]
	Nasal decongestants	7.15	[44-48]
	Cough suppressants e.g Codeine, Dextromethorphan	11.14	[49-53]
**One visit to A&E/one admission via the ER**	Visit to A&E/ER (Y/N)	992.02	[12, 13, 54-57]
**One night spent in hospital (not in ICU or A&E)**	Number of nights in hospital (not in ICU/A&E)	690.71	[58-63]
**One night spent in hospital (in ICU)**	Number of nights in hospital (in ICU)	2,192.75	[62-67]
**One test/assessment for RSV diagnosis**	RT-PCR	103.16	[17, 67, 68, 69]
	Rapid Antigen	31.07	[17, 67, 70, 71]
	Chest X-ray	31.30	[17, 67, 72, 73]
	Pulse oximetry	30.19	[17, 67, 74, 75]
**One test for suspected pneumococcal infection**	Urinalysis	13.02	[17, 67, 76, 77]
	Complete blood count (CBC)	12.42	[17, 67, 77, 78]
	Phlegm test	33.14	[17, 67, 79, 80]
	Chest X-ray	32.86	[15, 17, 67, 81]
**One additional procedure for an RSV patient**	Mechanical ventilation	8,766.17	N/A
	Intubation	352.08	[17, 82]
	Receiving oxygen/IV fluids	278.45	[15, 83]
	Receiving feeding tubes	481.19	[15, 17, 84]
	Lobectomy	2,040.05	[15, 85, 86]
	Nasal bulb suctioning	11.16	[87, 88]
**One hour patient earnings**	Adults >60 years old	18.29	[89-93]

Abbreviations: HCP – health care provider, OTC – over the counter

Supplementary Table S2: Physician demographics

Physician demographics	Overall	Geriatrician	PCP	Pulmonologist	Infectious disease specialist
**Country, n (%)**	**n=682**	**n=28**	**n=370**	**n=175**	**n=109**
France	127 (18.6%)	4 (14.3%)	85 (23.0%)	25 (14.3%)	13 (11.9%)
Germany	150 (22.0%)	4 (14.3%)	62 (16.8%)	50 (28.6%)	34 (31.2%)
Italy	135 (19.8%)	10 (35.7%)	48 (13.0%)	44 (25.1%)	33 (30.3%)
Spain	155 (22.7%)	5 (17.9%)	96 (25.9%)	31 (17.7%)	23 (21.1%)
United Kingdom	115 (16.9%)	5 (17.9%)	79 (21.4%)	25 (14.3%)	6 (5.5%)
**Years in practice, n (%)**					
<1 year	3 (0.4%)	0 (0.0%)	2 (0.5%)	1 (0.6%)	0 (0.0%)
1 – 2 years	6 (0.9%)	0 (0.0%)	3 (0.8%)	2 (1.1%)	1 (0.9%)
2 – 3 years	10 (1.5%)	0 (0.0%)	3 (0.8%)	2 (1.1%)	5 (4.6%)
3 – 4 years	24 (3.5%)	1 (3.5%)	7 (1.9%)	6 (3.4%)	10 (9.2%)

Confidential - Not for Public Consumption or Distribution

≥5 years	639 (93.7%)	27 (96.4%)	355 (95.9%)	164 (93.7%)	93 (85.3%)
**Setting majority of patients seen in, n (%)**					
Primary care	444 (65.1%)	10 (35.7%)	343 (92.7%)	58 (33.1%)	33 (30.3%)
Secondary care	238 (34.9%)	18 (64.3%)	27 (7.3%)	117 (66.9%)	76 (69.7%)

Abbreviations: PCP – primary care physician, SD – standard deviation

Supplementary Table S3: Resource utilization, caregiving needs and productivity loss – Acute - respiratory syncytial virus (A-RSV)

	**60+ years**		**60-74 years**			**75+ years**		
	**Without RF[1] (n=90)**	**With RF (n=147)**	**Total (n=188)**	**Without RF (n=79)**	**With RF (n=109)**	**Total (n=49†)**	**Without RF (n=11†)**	**With RF (n=38†)**
**Visited a PCP, n (%)**	75 (83.3%)	112 (76.2%)	143 (76.1%)	67 (84.8%)	76 (69.7%)	44 (89.8%)	8 (72.7%)	36 (94.7%)
**Visited a specialist[2], n (%)**	34 (37.8%)	66 (44.9%)	81 (43.1%)	28 (35.4%)	53 (48.6%)	19 (38.8%)	6 (54.5%)	13 (34.2%)
**Number of HCP visits per patient, mean (SD)**	2.9 (2.1)	3.2 (2.9)	2.7 (2.1)	2.8 (1.9)	2.7 (2.2)	4.5 (3.8)	3.7 (3.6)	4.7 (3.9)
PCP/ GP	0.3 (0.7)	0.4 (1.2)	0.3 (1.1)	0.2 (0.7)	0.4 (1.3)	0.3 (0.7)	0.7 (0.9)	0.2 (0.5)
Infectious disease specialist	0.3 (0.7)	0.4 (1.2)	0.3 (1.1)	0.2 (0.7)	0.4 (1.3)	0.3 (0.7)	0.7 (0.9)	0.2 (0.5)
Pulmonologist	0.2 (0.6)	0.4 (0.9)	0.4 (0.8)	0.2 (0.6)	0.4 (0.9)	0.3 (0.9)	0.2 (0.6)	0.3 (0.9)
Emergency physician	0.1 (0.2)	0.1 (0.3)	0.1 (0.2)	0.1 (0.3)	0.1 (0.2)	0.1 (0.3)	-	0.1 (0.3)
Geriatrician	0.0 (0.1)	0.1 (0.5)	0.0 (0.1)	0.0 (0.1)	0.0 (0.1)	0.2 (0.9)	-	0.2 (1.0)
**Number of tests (to diagnose or monitor) per patient, mean (SD)**	2.0 (1.4)	2.8 (2.2)	2.4 (1.7)	2.0 (1.3)	2.6 (1.9)	2.9 (2.6)	2.0 (1.6)	3.2 (2.8)
RT-PCR	0.6 (0.7)	0.9 (0.8)	0.9 (0.8)	0.6 (0.7)	1.0 (0.8)	0.7 (0.7)	0.5 (0.8)	0.7 (0.6)
Rapid antigen	0.7 (0.8)	0.5 (0.8)	0.6 (0.8)	0.7 (0.8)	0.5 (0.8)	0.8 (0.8)	1.0 (0.9)	0.7 (0.8)
Pulse oximetry	0.3 (0.7)	0.7 (1.3)	0.5 (0.9)	0.3 (0.7)	0.6 (1.0)	0.6 (1.8)	-	0.8 (2.1)
Chest x-ray	0.3 (0.5)	0.4 (0.6)	0.4 (0.6)	0.3 (0.5)	0.4 (0.6)	0.4 (0.6)	0.2 (0.4)	0.5 (0.7)
**Received prescribed medication (currently or previously), n (%)**	21 (23.3%)	53 (36.1%)	53 (28.2%)	19 (24.1%)	34 (31.2%)	21 (42.9%)	2 (18.2%)	19 (50.0%)
**Most recent medication prescribed, n (%)**	**n=21†**	**n=53**	**n=53**	**n=19†**	**n=34†**	**n=21†**	**n=2†**	**n=19†**
Ribavirin	12 (57.1%)	30 (56.6%)	31 (58.5%)	12 (63.2%)	19 (55.9%)	11 (52.4%)	0 (0.0%)	11 (57.9%)
Antibiotic	1 (4.8%)	3 (5.7%)	2 (3.8%)	1 (5.3%)	1 (2.9%)	2 (9.5%)	0 (0.0%)	2 (10.5%)
Corticosteroid	0 (0.0%)	1 (1.9%)	1 (1.9%)	0 (0.0%)	1 (2.9%)	0 (0.0%)	0 (0.0%)	0 (0.0%)
Analgesic	1 (4.8%)	2 (3.8%)	2 (3.8%)	0 (0.0%)	2 (5.9%)	1 (4.8%)	1 (50.0%)	0 (0.0%)
Other	7 (33.3%)	18 (34%)	17 (32.1%)	6 (31.6%)	11 (32.4%)	8 (38.1%)	1 (50.0%)	7 (36.8%)
**Over-the-counter medication taken, n (%)**	**n=90**	**n=147**	**n=188**	**n=79**	**n=109**	**n=49**	**n=11**	**n=38**
Paracetamol	44 (52.4%)	62 (49.6%)	73 (43.7%)	37 (50.7%)	36 (38.3%)	33 (78.6%)	7 (63.6%)	26 (83.9%)
Cough suppressants	40 (47.6%)	66 (52.8%)	92 (55.1%)	37 (50.7%)	55 (58.5%)	14 (33.3%)	3 (27.3%)	11 (35.5%)
Ibuprofen	40 (47.6%)	68 (54.4%)	95 (56.9%)	35 (47.9%)	60 (63.8%)	13 (31%)	5 (45.5%)	8 (25.8%)
Nasal decongestants	38 (45.2%)	51 (40.8%)	73 (43.7%)	33 (45.2%)	40 (42.6%)	16 (38.1%)	5 (45.5%)	11 (35.5%)
Other	7 (8.3%)	2 (1.6%)	6 (3.6%)	5 (6.8%)	1 (1.1%)	3 (7.1%)	2 (18.2%)	1 (3.2%)
**Received supportive care (currently), n (%)**	17 (18.9%)	58 (40%)	52 (27.8%)	16 (20.3%)	36 (33.3%)	23 (47.9%)	1 (9.1%)	22 (59.5%)
**Supportive care type, n (%)**	**n=17†**	**n=58**	**n=52**	**n=16†**	**n=36†**	**n=23†**	**n=1†**	**n=22†**
Non-professional[3]	17 (100.0%)	53 (91.4%)	52 (100.0%)	16 (100.0%)	36 (100.0%)	18 (78.3%)	1 (100.0%)	17 (77.3%)
Professional	0 (0.0%)	5 (8.6%)	0 (0.0%)	0 (0.0%)	0 (0.0%)	5 (21.7%)	0 (0.0%)	5 (22.7%)
**Total number of care hours received per week per patient, mean (SD)**	**n=8†**	**n=36†**	**n=33†**	**n=7†**	**n=26†**	**n=11†**	**n=1†**	**n=10†**
Non-professional	24.5 (11.2)	28.4 (33.3)	21.0 (11.7)	22.3 (10.1)	20.6 (12.2)	47.9 (54.3)	40.0 (0.0)	48.7 (57.2)
Professional	**n=0**	**n=3†**	**n=0**	**n=0**	**n=0**	**n=3†**	**n=0**	**n=3†**
	-	30.7 (16.2)	-	-	-	30.7 (16.2)	-	30.8 (16.2)
**.**	**n=4†**	**n=4†**	**n=4†**	**n=4†**	**n=4†**	**n=0**	**n=0**	**n=0**
**WPAI: percentage overall work impairment, mean (SD)**	48.2 (34.9)	48.2 (16.7)	48.2 (25.3)	48.2 (34.9)	48.2 (16.7)	-	-	-
**WPAI: percentage overall activity impairment, mean (SD)**	**n=43†**	**n=67**	**n=88**	**n=38†**	**n=50**	**n=22†**	**n=5†**	**n=17†**
	49.5 (22.5)	50.9 (22.8)	48.5 (21.9)	48.7 (23.6)	48.4 (20.7)	57.7 (24.3)	56.0 (8.9)	58.2 (27.4)
**Missed work due to RSV, n (%)**	5 (71.4%)	3 (60.0%)	8 (66.7%)	5 (71.4%)	3 (60.0%)	-	-	-
	**n=8†**	**n=26†**	**n=22†**	**n=8†**	**n=14†**	**n=12†**	**n=0**	**n=12†**

Confidential - Not for Public Consumption or Distribution

Missed hours of paid work due to RSV in the past seven days, mean (SD)	16.3 (17.0)	14.0 (15.9)	15.3 (15.8)	16.3 (17.0)	14.0 (15.9)	-	-	-
**Missed unpaid activity due to RSV, n (%)**	**n=40**	**n=61**	**n=82**	**n=36**	**n=46**	**n=18†**	**n-3†**	**n=15†**
	5 (44.4%)	9 (41.5%)	11 (43.6%)	5 (45.6%)	6 (14.3%)	3 (16.7%)	0 (0.0%)	3 (16.7%)
**Hours of unpaid activity lost per week, mean (SD)**	**n=5†**	**n=9†**	**n=11†**	**n=5†**	**n=6†**	**n=3†**	**n-0**	**n=3†**
	7.2 (3.9)	4.0 (3.3)	5.5 (4.0)	7.2 (3.9)	4.0 (3.5)	4.0 (2.8)	-	4.0 (2.8)
Supporting or caring for a family member	**n=1†**	**n=1†**	**n=1†**	**n=1†**	**n=0**	**n=1†**	**n=0**	**n=1†**
	6.0 (-)	6.0 (-)	6.0 (-)	6.0 (-)	- (-)	6.0 (-)	-	6.0 (-)
Supporting or caring for a friend	**n=0**	**n=0**	**n=0**	**n=0**	**n=0**	**n=0**	**n=0**	**n=0**
	-	-	-	-	-	-	-	-
Assisting in household activities	**n=1†**	**n=2†**	**n=2†**	**n=1†**	**n=1†**	**n=1†**	**n=0**	**n=1†**
	4.0 (-)	5.5 (0.7)	4.5 (0.7)	4 (-)	5 (-)	6 (-)	-	6 (-)
Voluntary work	**n=0**	**n=0**	**n=0**	**n=0**	**n=0**	**n=0**	**n=0**	**n=0**
	-	-	-	-	-	-	-	-
Other	**n=0**	**n=0**	**n=0**	**n=0**	**n=0**	**n=0**	**n=0**	**n=0**
	-	-	-	-	-	-	-	-

Answers of "Don't know" have been excluded from resource calculations. Across the total population, no more than 11.4% of physicians provided this answer for any one variable

Abbreviations: PCP – primary care physician, RF – risk factors, HCP – health care provider, SD – standard deviation, RT-PCR – reverse-transcription polymerase chain reaction, WPAI – work productivity and impairment scale

[1]Risk factors for severe RSV: Myocardial infarction, Diabetes with/without chronic complications, Renal disease, Any malignancy including leukemia and lymphoma, Metastatic solid tumor, Mild-severe liver disease, AIDS/HIV, Long term effects of COVID-19, Dementia, Chronic pulmonary disease, Asthma, Congestive heart failure, Pneumonia, Bronchiolitis, Bronchiectasis, Sickle cell anaemia, Severe anaemia, Thalassaemia, Cerebrovascular disease, Neutropenia, Chronic kidney disease (CKD), Stroke, Immunosuppression, Asthma and COPD (ACOS), Interstitial lung disease (ILD), Pulmonary embolism, Pulmonary hypertension, Cystic fibrosis, Coronary heart disease, Cardiomyopathy

[2]Specialist includes infectious disease specialist, pulmonologist, emergency physician, geriatrician

[3]Non-professional caregiver includes partner/spouse, parent/guardian, child under 18 years, child over 18 years, other relative(s), friend(s)/neighbor(s), other non-professional caregiver(s).

†Caution low base size

Supplementary Table S4: Resource utilization, caregiving needs and productivity loss – Ongoing - respiratory syncytial virus (O-RSV)

	**60+ years**		**60-74 years**			**75+ years**		
	**Without RF[1] (n=65)**	**With RF (n=131)**	**Total (n=144)**	**Without RF (n=60)**	**With RF (n=84)**	**Total (n=52)**	**Without RF (n=5†)**	**With RF (n=47†)**
**Visited a PCP/GP, n (%)**	56 (86.2%)	103 (78.6%)	118 (81.9%)	51 (85.0%)	67 (79.8%)	41 (78.8%)	5 (100.0%)	36 (76.6%)
**Visited a specialist[2], n (%)**	27 (41.5%)	70 (53.4%)	63 (43.8%)	23 (38.3%)	40 (47.6%)	34 (65.4%)	4 (80.0%)	30 (63.8%)
**Number of HCP visits per patient, mean (SD)**	3.3 (2.2)	4.2 (3.0)	3.5 (2.2)	3.2 (2.2)	3.7 (2.3)	5.1 (3.7)	4 (1.6)	5.2 (3.8)
PCP/ GP	2.4 (2.0)	3.1 (3.2)	2.5 (2.2)	2.4 (2.1)	2.6 (2.3)	3.9 (4.1)	2.2 (0.4)	4.1 (4.2)
Infectious disease specialist	0.4 (1.0)	0.6 (1.4)	0.5 (1.2)	0.4 (1.0)	0.5 (1.4)	0.6 (1.3)	-	0.7 (1.3)
Pulmonologist	0.4 (0.9)	0.5 (1.1)	0.4 (1.0)	0.3 (0.8)	0.5 (1.1)	0.6 (1.1)	1.6 (1.1)	0.5 (1.1)
Emergency physician	0.1 (0.3)	0.1 (0.3)	0.1 (0.4)	0.1 (0.3)	0.1 (0.4)	0.1 (0.3)	0.2 (0.4)	0.1 (0.3)
Geriatrician	-	0.1 (0.3)	-	-	-	0.1 (0.5)	-	0.1 (0.6)
**Number of tests (to diagnose or monitor) per patient, mean (SD)**	2.8 (2.2)	4.1 (4.2)	3.2 (2.4)	2.9 (2.2)	3.4 (2.5)	5.0 (5.7)	2.4 (2.1)	5.3 (6.0)
RT-PCR	0.7 (0.7)	1.0 (1.0)	0.8 (0.8)	0.8 (0.7)	0.8 (0.8)	1.3 (1.2)	0.4 (0.6)	1.3 (1.1)
Rapid antigen	0.9 (0.9)	0.9 (1.5)	0.8 (0.9)	0.9 (0.9)	0.8 (1.0)	1.0 (2.1)	1.2 (0.8)	1.0 (2.2)
Pulse oximetry	0.6 (1.2)	1.0 (1.8)	0.7 (1.4)	0.6 (1.2)	0.8 (1.6)	1.1 (2.0)	0.4 (0.9)	1.2 (2.1)
Chest x-ray	0.4 (0.7)	0.6 (0.7)	0.5 (0.7)	0.4 (0.7)	0.6(0.7)	0.48 (0.7)	0.4 (0.9)	0.5 (0.7)
**Received prescribed medication (currently or previously), n (%)**	20 (31.7%)	51 (38.9%)	49 (34.5%)	17 (29.3%)	32 (38.1%)	22 (42.3%)	3 (60.0%)	19 (40.4%)
**Most recent medication prescribed, n (%)**	**n=20†**	**n=51**	**n=49†**	**n=17†**	**n=32†**	**n=22†**	**n=3†**	**n=19†**
Ribavirin	17 (85.0%)	28 (54.9%)	29 (59.2%)	14 (82.4%)	15 (46.9%)	16 (72.7%)	3 (100.0%)	13 (68.4%)
Antibiotic	0 (0.0%)	3 (5.9%)	2 (4.1%)	0 (0.0%)	2 (6.3%)	1 (4.5%)	0 (0.0%)	1 (5.3%)
Corticosteroid	0 (0.0%)	2 (3.9%)	1 (2.0%)	0 (0.0%)	1 (3.1%)	1 (4.5%)	0 (0.0%)	1 (5.3%)
Analgesic	0 (0.0%)	1 (2.0%)	1 (2.0%)	0 (0.0%)	1 (3.1%)	0 (0.0%)	0 (0.0%)	0 (0.0%)
Other	7 (33.3%)	18 (34%)	17 (32.1%)	6 (31.6%)	11 (32.4%)	8 (38.1%)	1 (50.0%)	7 (36.8%)
**Over-the-counter medication taken, n (%)**	**n=65**	**n=131**	**n=144**	**n=60**	**n=84**	**n=52**	**n=5†**	**n=47†**
Paracetamol	30 (55.6%)	72 (65.5%)	69 (59%)	25 (51%)	44 (64.7%)	33 (70.2%)	5 (100%)	28 (66.7%)
Cough suppressants	14 (25.9%)	47 (42.7%)	49 (41.9%)	14 (28.6%)	35 (51.5%)	12 (25.5%)	0 (0.0%)	12 (28.6%)
Ibuprofen	26 (48.1%)	39 (35.5%)	46 (39.3%)	22 (44.9%)	24 (35.3%)	19 (40.4%)	4 (80.0%)	15 (35.7%)
Nasal decongestants	14 (25.9%)	29 (26.4%)	32 (27.4%)	13 (26.5%)	19 (27.9%)	11 (23.4%)	1 (20.0%)	10 (23.8%)
Other	5 (9.3%)	7 (6.4%)	10 (8.5%)	5 (10.2%)	5 (7.4%)	2 (4.3%)	0 (0.0%)	2 (4.8%)

Confidential - Not for Public Consumption or Distribution

**Received supportive care (currently), n (%)**	10 (15.6%)	53 (43.1%)	27 (19.9%)	7 (11.9%)	20 (26.0%)	36 (70.6%)	3 (60.0%)	33 (71.7%)
**Supportive care type, n (%)**	**n=10†**	**n=53**	**n=27†**	**n=7†**	**n=20†**	**n=36†**	**n=3†**	**n=33†**
Non-professional[3]	10 (100.0%)	52 (98.1%)	27 (100.0%)	7 (100.0%)	20 (100.0%)	35 (97.2%)	3 (100.0%)	32 (97.0%)
Professional	0 (0.0%)	7 (13.2%)	0 (0.0%)	0 (0.0%)	0 (0.0%)	7 (19.4%)	0 (0.0%)	7 (21.2%)
**Total number of care hours per week per patient, mean (SD)**	**n=6†**	**n=33†**	**n=17†**	**n=4†**	**n=13†**	**n=22†**	**n=2†**	**n=20†**
Non-professional	20.8 (19.0)	23.7 (25.8)	19.4 (14.0)	16.3 (15.9)	20.3 (13.9)	26.3 (30.6)	30.0 (28.3)	25.9 (31.5)
Professional	**n=0**	**n=5†**	**n=0**	**n=0**	**n=0**	**n=5†**	**n=0**	**n=5†**
	-	20.6 (14.7)	-	-	-	20.6 (14.7)	-	20.6 (14.7)
**WPAI: percentage overall work impairment, mean (SD)**	**n=5†**	**n=1†**	**n=6†**	**n=5†**	**n=1†**	-	-	-
	62.6 (19.0)	-	52.1 (30.7)	62.6 (19.0)	-	-	-	-
**WPAI: percentage overall activity impairment, mean (SD)**	**n=13†**	**n=30†**	**n=34†**	**n=12†**	**n=22†**	**n=9†**	**n=1†**	**n=8†**
	51.5 (24.8)	45.7 (27.8)	44.1 (27.3)	51.7(25.9)	40.0 (27.8)	60 (21.2)	50.0 (0.0)	61.3 (22.3)
**Missed work due to RSV, n (%)**	**n=6†**	**n=1†**	**n=7†**	**n=6†**	**n=1†**	**n=0**	**n=0**	**n=0**
	6 (100.0%)	0 (0.0%)	6 (85.7%)	6 (100.0%)	0 (0.0%)	-	-	-
Missed hours of paid work due to RSV in the past seven days, mean (SD)	18.3 (9.2)	0.0 (-)	15.7 (10.9)	18.3 (9.2)	0.0 (-)	-	-	-
**Missed unpaid activity due to RSV, n (%)**	**n=11†**	**n=29†**	**n=32†**	**n=10†**	**n=22†**	**n=7†**	**n=1†**	**n=6†**
	2 (18.2%)	3 (10.3%)	2 (6.2%)	0 (0.0%)	2 (9.1%)	1 (14.3%)	0 (0.0%)	1 (16.7%)
	**n=2†**	**n=3†**	**n=2†**	**n=0**	**n=2†**	**n=1†**	**n=0**	**n=1†**
**Hours of unpaid activity lost per week, mean (SD)**	12.0 (12.0)	6.0 (5.5)	8.0 (9.5)	-	5.3 (14.0)	8 (0.0)	0 (0.0)	8 (0.0)
Supporting or caring for a family member	**n=0**	**n=1†**	**n=1†**	**n=0**	**n=1†**	**n=0**	**n=0**	**n=0**
	-	2.0 (-)	2.0 (-)	-	2.0 (-)	-	-	-
Supporting or caring for a friend	**n=0**	**n=1†**	**n=0**	**n=0**	**n=0**	**n=1†**	**n=0**	**n=1†**
	-	8.0 (-)	-	-	-	8.0 (-)	-	8.0 (-)
Assisting in household activities	**n=0**	**n=0**	**n=0**	**n=0**	**n=0**	**n=0**	**n=0**	**n=0**
	-	-	-	-	-	-	-	-
Voluntary work	**n=0**	**n=0**	**n=0**	**n=0**	**n=0**	**n=0**	**n=0**	**n=0**
	-	-	-	-	-	-	-	-
Other	**n=0**	**n=1†**	**n=1†**	**n=0**	**n=1†**	**n=0**	**n=0**	**n=0**
	-	14.0 (-)	14.0 (-)	-	14.0 (-)	-	-	-

Answers of "Don't know" have been excluded from resource calculations. Across the total population, no more than 11.4% of physicians provided this answer for any one variable

Abbreviations: PCP – primary care physician, RF – risk factors, HCP – health care provider, SD – standard deviation, RT-PCR – reverse-transcription polymerase chain reaction, WPAI – work productivity and impairment scale

[1]Risk factors for severe RSV: Myocardial infarction, Diabetes with/without chronic complications, Renal disease, Any malignancy including leukemia and lymphoma, Metastatic solid tumor, Mild-severe liver disease, AIDS/HIV, Long term effects of COVID-19, Dementia, Chronic pulmonary disease, Asthma, Congestive heart failure, Pneumonia, Bronchiolitis, Bronchiectasis, Sickle cell anaemia, Severe anaemia, Thalassaemia, Cerebrovascular disease, Neutropenia, Chronic kidney disease (CKD), Stroke, Immunosuppression, Asthma and COPD (ACOS), Interstitial lung disease (ILD), Pulmonary embolism, Pulmonary hypertension, Cystic fibrosis, Coronary heart disease, Cardiomyopathy

[2]Specialist includes infectious disease specialist, pulmonologist, emergency physician, geriatrician

[3]Non-professional caregiver includes partner/spouse, parent/guardian, child under 18 years, child over 18 years, other relative(s), friend(s)/neighbor(s), other non-professional caregiver(s).

†Caution low base size

Supplementary Table S5: Resource utilization, caregiving needs and productivity loss – Post-acute - respiratory syncytial virus (P-RSV)

	**60+ years**		**60-74 years**			**75+ years**		
	**Without RF[1] (n=170)**	**With RF (n=418)**	**Total (n=401)**	**Without RF (n=125)**	**With RF (n=276)**	**Total (n=187)**	**Without RF (n=45)**	**With RF (n=142)**
**Visited a PCP/GP, n (%)**	149 (87.6%)	349 (83.5%)	331 (82.5%)	110 (88%)	221 (80.1%)	167 (89.3%)	39 (86.7%)	128 (90.1%)
**Visited a specialist[2], n (%)**	66 (38.8%)	227 (54.3%)	193 (48.1%)	48 (38.4%)	145 (52.5%)	100 (53.5%)	18 (40%)	82 (57.7%)
**Number of HCP visits per patient, mean (SD)**	4.2 (2.7)	4.8 (3.3)	4.1 (2.8)	3.8 (2.2)	4.3 (3.0)	5.6 (3.6)	5.5 (3.5)	5.6 (3.6)
PCP/ GP	3.4 (2.6)	3.8 (3.6)	3.2 (3.0)	3 (2.2)	3.3 (3.4)	4.7 (3.6)	4.4 (3.4)	4.7 (3.7)
Infectious disease specialist	0.5 (1.2)	0.4 (1.1)	0.5 (1.2)	0.5 (1.2)	0.5 (1.2)	0.3 (1.0)	0.5 (1.2)	0.3 (0.9)
Pulmonologist	0.4 (0.9)	0.7 (1.3)	0.6 (1.2)	0.4 (0.9)	0.8 (1.4)	0.5 (1.2)	0.4 (1.0)	0.6 (1.2)
Emergency physician	0.1 (0.3)	0.1 (0.5)	0.1 (0.4)	0.1 (0.3)	0.1 (0.4)	0.2 (0.5)	0.1 (0.3)	0.2 (0.5)
Geriatrician	0.0 (0.2)	0.1 (0.5)	0.0 (0.2)	0.0 (0.1)	0.0 (0.2)	0.1 (0.7)	0.1 (0.5)	0.1 (0.8)

Confidential - Not for Public Consumption or Distribution

**Number of tests (to diagnose or monitor) per patient, mean (SD)**	3.7 (4.7)	5.6 (6.2)	4.7 (4.9)	3.5 (2.7)	5.3 (5.5)	5.7 (7.5)	4.4 (7.9)	6.1 (7.4)
RT-PCR	1.0 (1.0)	1.2 (1.3)	1.2 (1.2)	1.0 (1.0)	1.3 (1.2)	1.0 (1.2)	0.8 (0.9)	1.0 (1.3)
Rapid antigen	1.1 (1.2)	1.0 (1.5)	1.0 (1.4)	1.1 (1.3)	0.9 (1.5)	1.1 (1.4)	1.1 (1.1)	1.1 (1.4)
Pulse oximetry	0.7 (1.5)	1.3 (2.4)	1.0 (1.9)	0.7 (1.4)	1.1 (2.1)	1.3 (2.7)	0.6 (1.7)	1.6 (2.9)
Chest x-ray	0.5 (0.8)	0.9 (0.9)	0.8 (0.9)	0.5 (0.8)	0.9 (0.9)	0.8 (0.9)	0.4 (0.8)	0.9 (0.9)
**Received prescribed medication (currently or previously), n (%)**	56 (33.5%)	155 (37.9%)	128 (32.6%)	35 (28.7%)	93 (34.3%)	83 (45.4%)	21 (46.7%)	62 (44.9%)
**Most recent medication prescribed, n (%)**	**n=56**	**n=155**	**n=128**	**n=35†**	**n=93**	**n=83**	**n=21†**	**n=62**
Ribavirin	33 (58.9%)	89 (57.4%)	78 (60.9%)	24 (68.6%)	54 (58.1%)	44 (53.0%)	9 (42.9%)	35 (56.5%)
Antibiotic	2 (3.6%)	2 (1.3%)	1 (0.8%)	0 (0.0%)	1 (1.1%)	3 (3.6%)	2 (9.5%)	1 (1.6%)
Corticosteroid	0 (0.0%)	0 (0.0%)	0 (0.0%)	0 (0.0%)	0 (0.0%)	0 (0.0%)	0 (0.0%)	0 (0.0%)
Analgesic	3 (5.4%)	3 (1.9%)	4 (3.1%)	2 (5.7%)	2 (2.2%)	2 (2.4%)	1 (4.8%)	1 (1.6%)
Other	18 (32.1%)	61 (39.4%)	45 (35.2%)	9 (25.7%)	36 (38.7%)	34 (41.0%)	9 (42.9%)	25 (40.3%)
**Over-the-counter medication taken, n (%)**	**n=170**	**n=418**	**n=401**	**n=125**	**n=276**	**n=187**	**n=45**	**n=142**
Paracetamol	80 (64.5%)	203 (59.9%)	179 (55.6%)	56 (62.2%)	123 (53%)	104 (73.8%)	24 (70.6%)	80 (74.8%)
Cough suppressants	44 (35.5%)	149 (44.0%)	142 (44.1%)	36 (40.0%)	106 (45.7%)	51 (36.2%)	8 (23.5%)	43 (40.2%)
Ibuprofen	49 (39.5%)	155 (45.7%)	160 (49.7%)	38 (42.2%)	122 (52.6%)	44 (31.2%)	11 (32.4%)	33 (30.8%)
Nasal decongestants	33 (26.6%)	109 (32.2%)	102 (31.7%)	26 (28.9%)	76 (32.8%)	40 (28.4%)	7 (20.6%)	33 (30.8%)
Other	8 (6.5%)	18 (5.3%)	17 (5.3%)	6 (6.7%)	11 (4.7%)	9 (6.4%)	2 (5.9%)	7 (6.5%)
**Received supportive care (currently), n (%)**	43 (25.6%)	148 (36.4%)	98 (24.9%)	27 (21.8%)	71 (26.4%)	93 (51.1%)	16 (36.4%)	77 (55.8%)
**Caregiver type, n (%)**	**n=43**	**n=148**	**n=98**	**n=27†**	**n=71**	**n=93**	**n=16†**	**n=77**
Non-professional[3]	43 (100.0%)	136 (91.9%)	98 (100.0%)	27 (100.0%)	71 (100%)	81 (87.1%)	16 (100.0%)	65 (84.4%)
Professional	0 (0.0%)	20 (13.5%)	0 (0.0%)	0 (0.0%)	0 (0.0%)	20 (21.5%)	0 (0.0%)	20 (26.0%)
**Total number of care hours per week per patient, mean (SD)**	**n=26†**	**n=78**	**n=62**	**n=17†**	**n=45†**	**n=42†**	**n=9†**	**n=33†**
Non-professional	30.3 (41.2)	30.1 (36.0)	24.8 (34.8)	23.5 (39.4)	25.3 (33.3)	38.0 (39.5)	43.2 (43.8)	36.6 (38.9)
Professional	**n=0**	**n=15†**	**n=0**	**n=0**	**n=0**	**n=15†**	**n=0**	**n=15†**
	-	47.3 (50.3)	-	-	-	47.3 (50.2)	-	47.3 (50.3)
**WPAI: percentage overall work impairment, mean (SD)**	**n=1**	**n=9**	**n=10**	**n=1**	**n=9**	**n=0**	**n=0**	**n=0**
	30.0 (0.0)	60.2 (30.8)	57.2 (30.5)	30.0 (0.0)	60.2 (30.8)	-	-	-
**WPAI: percentage overall activity impairment, mean (SD)**	**n=39†**	**n=84**	**n=92**	**n=27†**	**n=65**	**n=31†**	**n=12†**	**n=19†**
	50.0 (27.1)	44.9 (23.0)	44.5 (24.1)	45.9 (26.1)	43.9 (23.4)	52.6 (24.6)	59.2 (28.1)	48.4 (21.9)
**Missed work due to RSV, n (%)**	**n=6†**	**n=12†**	**n=7†**	**n=6†**	**n=12†**	**n=0**	**n=0**	**n=0**
	6 (100.0%)	0 (0.0%)	6 (85.7%)	6 (100.0%)	0 (0.0%)	-	-	-
Missed hours of paid work due to RSV in the past seven days, mean (SD)	18.3 (9.2)	0.0 (-)	15.7 (10.9)	18.3 (9.2)	0.0 (0.0)	-	-	-
**Missed unpaid activity due to RSV, n (%)**	**n=34†**	**n=80**	**n=87**	**n=25†**	**n=62**	**n=22†**	**n=4†**	**n=18†**
	3 (8.8%)	10 (12.5%)	11 (12.6%)	3 (12.0%)	8 (12.9%)	2 (9.1%)	0 (0.0%)	2 (11.1%)
	**n=3†**	**n=10†**	**n=11†**	**n=3†**	**n=8†**	**n=2†**	**n=0**	**n=2†**
**Hours of unpaid activity lost per week, mean (SD)**	4.0 (2.2)-	10.3 (10.9)	9.5 (10.5)	4.0 (2.2)	11.6 (11.6)	5.0 (5.0)	-	5.0 (5.0)
Supporting or caring for a family member	**n=0**	**n=4†**	**n=3†**	**n=0**	**n=3†**	**n=1†**	**n=0**	**n=1†**
	-	16.5 (15.8)	18.7 (18.6)	-	18.7 (18.6)	10.0 (-)	-	10.0 (-)
Supporting or caring for a friend	**n=0**	**n=0**	**n=0**	**n=0**	**n=0**	**n=0**	**n=0**	**n=0**
	-	-	-	-	-	-	-	-
Assisting in household activities	**n=2†**	**n=1†**	**n=3†**	**n=2†**	**n=1†**	**n=0**	**n=0**	**n=0**
	5.0 (2.8)	2 (-)	4.0 (2.7)	5.0 (2.8)	2.0 (-)	-	-	-
Voluntary work	**n=0**	**n=0**	**n=0**	**n=0**	**n=0**	**n=0**	**n=0**	**n=0**
	-	-	-	-	-	-	-	-
Other	**n=0**	**n=0**	**n=0**	**n=0**	**n=0**	**n=0**	**n=0**	**n=0**
	-	-	-	-	-	-	-	-

Answers of "Don't know" have been excluded from resource calculations. Across the total population, no more than 11.4% of physicians provided this answer for any one variable

Abbreviations: PCP – primary care physician, RF – risk factors, HCP – health care provider, SD – standard deviation, RT-PCR – reverse-transcription polymerase chain reaction, WPAI – work productivity and impairment scale

[1]Risk factors for severe RSV: Myocardial infarction, Diabetes with/without chronic complications, Renal disease, Any malignancy including leukemia and lymphoma, Metastatic solid tumor, Mild-severe liver disease, AIDS/HIV, Long term effects of COVID-19, Dementia, Chronic pulmonary disease, Asthma, Congestive heart failure, Pneumonia, Bronchiolitis, Bronchiectasis, Sickle cell anemia, Severe anemia, Thalassemia, Cerebrovascular disease, Neutropenia, Chronic kidney disease (CKD), Stroke, Immunosuppression, Asthma and COPD

Confidential - Not for Public Consumption or Distribution

(ACOS), Interstitial lung disease (ILD), Pulmonary embolism, Pulmonary hypertension, Cystic fibrosis, Coronary heart disease, Cardiomyopathy
[2]Specialist includes infectious disease specialist, pulmonologist, emergency physician, geriatrician
[3]Non-professional caregiver includes partner/spouse, parent/guardian, child under 18 years, child over 18 years, other relative(s), friend(s)/neighbor(s), other non-professional caregiver(s).
†Caution low base size

Supplementary Table S6: Resource utilization, caregiving needs and productivity loss – Hospitalized - respiratory syncytial virus (H-RSV)

	60+ years		60-74 years			75+ years		
	**Without RF[1] (n=79)**	**With RF (n=481)**	**Total (n=369)**	**Without RF (n=59)**	**With RF (n=310)**	**Total (n=191)**	**Without RF (n=20†)**	**With RF (n=171)**
**Visited a PCP/GP, n (%)**	41 (51.9%)	259 (53.8%)	200 (54.2%)	33 (55.9%)	167 (53.9%)	100 (52.4%)	8 (40.0%)	92 (53.8%)
**Visited a specialist[2], n (%)**	76 (96.2%)	461 (95.8%)	353 (95.7%)	56 (94.9%)	297 (95.8%)	184 (96.3%)	20 (100.0%)	164 (95.9%)
**Number of HCP visits per patient, mean (SD)**	4.6 (4.9)	4.6 (3.6)	4.4 (3.1)	4.3 (3.9)	4.4 (2.9)	5 (4.9)	5.2 (7.1)	5 (4.6)
PCP/ GP	1.2 (1.9)	1.5 (2.9)	1.3 (2.3)	1.3 (1.8)	1.3 (2.4)	1.7 (3.5)	1.1 (2.0)	1.8 (3.7)
Infectious disease specialist	1.5 (4.7)	1 (2.2)	0.9 (2.2)	1.3 (3.8)	0.9 (1.8)	1.4 (3.3)	2.2 (6.6)	1.4 (2.7)
Pulmonologist	1.4 (1.8)	1.7 (2.4)	1.8 (2.3)	1.4 (1.9)	1.9 (2.4)	1.3 (2.3)	1.2 (1.4)	1.4 (2.4)
Emergency physician	0.6 (0.8)	0.4 (0.7)	0.4 (0.7)	0.5 (0.7)	0.4 (0.7)	0.6 (0.7)	0.9 (1.0)	0.5 (0.6)
Geriatrician	0.1 (0.4)	0.3 (1.0)	0.2 (1.0)	0.1 (0.5)	0.2 (1.0)	0.3 (0.9)	0.1 (0.2)	0.3 (1.0)
**Number of tests (to diagnose or monitor) per patient, mean (SD)**	7.2 (7.0)	10.2 (11.8)	8.6 (7.6)	6.7 (5.8)	9.0 (7.8)	11.9 (16.1)	8.7 (9.8)	12.3 (16.7)
RT-PCR	1.0 (0.9)	1.4 (0.9)	1.4 (1.0)	1.0 (0.9)	1.4 (1.0)	1.2 (0.8)	1.1 (0.8)	1.2 (0.8)
Rapid antigen	0.7 (1.1)	0.8 (1.3)	0.9 (1.4)	0.8 (1.0)	0.9 (1.4)	0.6 (1.0)	0.6 (1.4)	0.6 (0.9)
Pulse oximetry	1.1 (2.8)	1.4 (2.4)	1.3 (2.6)	1.3 (3.2)	1.3 (2.4)	1.4 (2.4)	0.6 (1.1)	1.5 (2.5)
Chest x-ray	1.2 (1.0)	1.6 (1.2)	1.5 (1.1)	1.1 (1.0)	1.6 (1.2)	1.7 (1.3)	1.4 (1.1)	1.7 (1.3)
**Received prescribed medication (currently or previously), n (%)**	38 (49.4%)	265 (55.7%)	217 (59.9%)	29 (50.9%)	188 (61.6%)	86 (45.0%)	9 (45.0%)	77 (45.0%)
**Most recent medication prescribed, n (%)**	**n=38†**	**n=265**	**n=217**	**n=29†**	**n=188**	**n=86**	**n=9†**	**n=77**
Ribavirin	27 (71.1%)	181 (68.3%)	150 (69.1%)	19 (65.5%)	131 (69.7%)	58 (67.4%)	8 (88.9%)	50 (64.9%)
Antibiotic	7 (18.4%)	26 (9.8%)	25 (11.5%)	7 (24.1%)	18 (9.6%)	8 (9.3%)	0 (0.0%)	8 (10.4%)
Corticosteroid	0 (0.0%)	11 (4.2%)	8 (3.7%)	0 (0.0%)	8 (4.3%)	3 (3.5%)	0 (0.0%)	3 (3.9%)
Analgesic	0 (0.0%)	1 (0.4%)	0 (0.0%)	0 (0.0%)	0 (0.0%)	1 (1.2%)	0 (0.0%)	1 (1.3%)
Other	4 (10.5%)	50 (18.9%)	36 (16.6%)	3 (10.3%)	33 (17.6%)	18 (20.9%)	1 (11.1%)	17 (22.1%)
**Over-the-counter medication taken, n (%)**	**n=79**	**n=481**	**n=369**	**n=59**	**n=310**	**n=191**	**n=20†**	**n=171**
Paracetamol	30 (55.6%)	290 (74.7%)	196 (67.8%)	22 (53.7%)	174 (70.2%)	124 (81.0%)	8 (61.5%)	116 (82.9%)
Cough suppressants	11 (20.4%)	150 (38.7%)	108 (37.4%)	6 (14.6%)	102 (41.1%)	53 (34.6%)	5 (38.5%)	48 (34.3%)
Ibuprofen	20 (37.0%)	110 (28.4%)	91 (31.5%)	16 (39.0%)	75 (30.2%)	39 (25.5%)	4 (30.8%)	35 (25.0%)
Nasal decongestants	11 (20.4%)	104 (26.8%)	74 (25.6%)	9 (22.0%)	65 (26.2%)	41 (26.8%)	2 (15.4%)	39 (27.9%)
Other	4 (7.4%)	51 (13.1%)	31 (10.7%)	3 (7.3%)	28 (11.3%)	24 (15.7%)	1 (7.7%)	23 (16.4%)
**Received supportive care (currently), n (%)**	17 (22.1%)	223 (47.2%)	141 (39.0%)	11 (19.3%)	130 (42.6%)	99 (52.9%)	6 (30.0%)	93 (55.7%)
**Supportive care type, n (%)**	**n=17†**	**n=223**	**n=141**	**n=11†**	**n=130**	**n=99**	**n=6†**	**n=93**
Non-professional[3]	16 (94.1%)	212 (95.1%)	138 (97.9%)	10 (90.9%)	128 (98.5%)	90 (90.9%)	6 (100.0%)	84 (90.3%)
Professional	1 (5.9%)	25 (11.2%)	8 (5.7%)	1 (9.1%)	7 (5.4%)	18 (18.2%)	0 (0.0%)	18 (19.4%)
**Total number of care hours per week per patient, mean (SD)**	**n=9†**	**n=98**	**n=75**	**n=6†**	**n=69**	**n=32†**	**n=3†**	**n=29†**
Non-professional	22.8 (20.0)	33.9 (34.9)	27.0 (27.1)	16.5 (16.9)	27.9 (27.7)	47 (43.7)	35.3 (23.2)	48.2 (45.4)
Professional	**n=1†**	**n=18†**	**n=6†**	**n=1†**	**n=5†**	**n=13†**	**n=0**	**n=13†**
	20.0 (-)	35.3 (43.8)	10.5 (5.8)	20.0 (-)	8.6 (3.9)	45.6 (47.9)	-	45.6 (47.0)
**Hospitalization**	**n=56**	**n=396**	**n=293**	**n=41**	**n=252**	**n=159**	**n=15†**	**n=144**
**Discharged on same day as admission, n (%)**	6 (9.8%)	29 (7.0%)	26 (8.4%)	5 (10.9%)	21 (7.9%)	9 (5.4%)	1 (6.7%)	8 (5.3%)
**Requiring one or more overnight stays, n (%)**	50 (82.0%)	367 (88.2%)	267 (85.9%)	36 (78.3%)	231 (87.2%)	150 (90.4%)	14 (93.3%)	136 (90.1%)
Total number of nights spent in hospital, mean (SD)	6.1 (4.6)	7.3 (5.5)	6.4 (4.1)	5.3 (3.5)	6.5 (4.2)	8.6 (7.1)	7.9 (6.4)	8.7 (7.1)
	**n=46**	**n=303**	**n=217**	**n=33†**	**n=184**	**n=132**	**n=13†**	**n=119**

Confidential - Not for Public Consumption or Distribution

Total number of nights spent in hospital (patients never admitted to ICU), mean (SD)	5.8 (4.1)	6.7 (5.3)	5.8 (3.5)	5.3 (3.6)	5.9 (3.5)	7.9(6.9)	6.9 (5.2)	8.0 (7.0)
Total number of nights spent in hospital (patients admitted to ICU), mean (SD)	**n=4†**	**n=63**	**n=50**	**n=3†**	**n=47**	**n=17**	**n=1†**	**n=16†**
	9.5 (8.5)	10.2(5.8)	8.9 (5.3)	5.3 (2.1)	9.1 (5.4)	13.7 (6.3)	22.0 (-)	13.3 (6.2)
**Admitted to ICU, n (%)**	4 (6.6%)	65 (15.6%)	50 (16.1%)	3 (6.5%)	47 (17.7%)	19 (11.4%)	1 (6.7%)	18 (11.9%)
Nights spent in ICU, mean (SD) (of those admitted to ICU)	2.8 (1.0)	3.3 (2.1)	3.1 (2.1)	2.3 (0.6)	3.2 (2.1)	3.7 (1.8)	4.0 (0.0)	3.6 (1.9)
**Received procedures for most recent infection while hospitalized, n (%)**	51 (65.4%)	364 (77.1%)	263 (73.1%)	36 (62.1%)	227 (75.2%)	152 (80.0%)	15 (75.0%)	137 (80.6%)
**Procedures received for most recent infection while hospitalized, n (%)**	**n=51**	**n=364**	**n=263**	**n=36†**	**n=227**	**n=152**	**n=15†**	**n=137**
Receiving oxygen/IV fluids	51 (100%)	348 (95.6%)	252 (95.8%)	36 (100.0%)	216 (95.2%)	147 (96.7%)	15 (100.0%)	132 (96.4%)
Nasal bulb suctioning	5 (9.8%)	49 (13.5%)	26 (9.9%)	3 (8.3%)	23 (10.1%)	28 (18.4%)	2 (13.3%)	26 (19%)
Mechanical ventilation	3 (5.9%)	41 (11.3%)	31 (11.8%)	3 (8.3%)	28 (12.3%)	13 (8.6%)	0 (0.0%)	13 (9.5%)
Receiving feeding tubes	2 (3.9%)	18 (4.9%)	9 (3.4%)	2 (5.6%)	7 (3.1%)	11 (7.2%)	0 (0.0%)	11 (8.0%)
Intubation	1 (2.0%)	12 (3.3%)	7 (2.7%)	1 (2.8%)	6 (2.6%)	6 (3.9%)	0 (0.0%)	6 (4.4%)
**WPAI: percentage overall work impairment, mean (SD)**	**n=1**	**n=4**	**n=5**	**n=1**	**n=4**	**n=0**	**n=0**	**n=0**
	20.0 (0.0)	61.7 (20.8)	53.3 (25.9)	20.0 (-)	61.7 (20.8)	-	-	-
**WPAI: percentage overall activity impairment, mean (SD)**	**n=26†**	**n=103**	**n=100**	**n=21†**	**n=79**	**n=29†**	**n=5†**	**n=24†**
	59.2 (23.0)	57.6 (25.3)	56.8 (23.8)	60.0 (20.0)	56.0 (24.8)	61.7 (27.9)	56.0 (35.8)	62.9 (26.9)
**Missed work due to RSV, n (%)**	**n=6†**	**n=13†**	**n=19†**	**n=6†**	**n=13†**	**n=0**	**n=0**	**n=0**
	5 (83.3%)	10 (76.9%)	15 (78.9%)	5 (83.3%)	10 (76.9%)	-	-	-
Missed hours of paid work due to RSV in the past seven days, mean (SD)	24.1 (14.1)	30.2 (21.9)	28.2 (19.6)	24.1 (14.1)	30.2 (21.9)	-	-	-
**Missed unpaid activity due to RSV, n (%)**	**n=22†**	**n=94**	**n=91**	**n=17†**	**n=74**	**n=25†**	**n=5†**	**n=20†**
	8 (36.4%)	26 (27.6%)	29 (31.8%)	7 (41.2%)	22 (29.7%)	5 (20.0%)	4 (80.0%)	1 (5.0%)
	**n=8†**	**n=26†**	**n=29†**	**n=7†**	**n=22†**	**n=5†**	**n=4†**	**n=1†**
**Hours of unpaid activity lost per week, mean (SD)**	7.8 (6.7)	5.6 (6.9)	6.7 (7.3)	8.3 (7.0)	6.3 (7.3)	2.4 (1.6)	2.0 (1.6)	1 (5.0%)
Supporting or caring for a family member	**n=2†**	**n=2†**	**n=4†**	**n=2†**	**n=2†**	**n=0**	**n=0**	**n=0**
	12.0 (11.3)	12.5 (3.5)	12.3 (6.9)	12 (11.3)	12.5 (3.5)	-	-	-
Supporting or caring for a friend	**n=0**	**n=0**	**n=0**	**n=0**	**n=0**	**n=0**	**n=0**	**n=0**
	-	-	-	-	-	-	-	-
Assisting in household activities	**n=0**	**n=13†**	**n=11†**	**n=0**	**n=11†**	**n=2†**	**n=0**	**n=2†**
	-	7.9 (5.7)	8.9 (5.5)	-	8.9 (5.5)	2.0 (1.4)	-	2.0 (1.4)
Voluntary work	**n=0**	**n=1†**	**n=1†**	**n=0**	**n=1†**	**n=0**	**n=0**	**n=0**
	-	4.0 (-)	4.0 (-)	-	4.0 (-)	-	-	-
Other	**n=0**	**n=0**	**n=0**	**n=0**	**n=0**	**n=0**	**n=0**	**n=0**
	-	-	-	-	-	-	-	-

Answers of "Don't know" have been excluded from resource calculations. Across the total population, no more than 11.4% of physicians provided this answer for any one variable

Abbreviations: PCP – primary care physician, RF – risk factors, HCP – health care provider, SD – standard deviation, RT-PCR – reverse-transcription polymerase chain reaction, WPAI – work productivity and impairment scale

[1]Risk factors for severe RSV: Myocardial infarction, Diabetes with/without chronic complications, Renal disease, Any malignancy including leukemia and lymphoma, Metastatic solid tumor, Mild-severe liver disease, AIDS/HIV, Long term effects of COVID-19, Dementia, Chronic pulmonary disease, Asthma, Congestive heart failure, Pneumonia, Bronchiolitis, Bronchiectasis, Sickle cell anemia, Severe anemia, Thalassemia, Cerebrovascular disease, Neutropenia, Chronic kidney disease (CKD), Stroke, Immunosuppression, Asthma and COPD (ACOS), Interstitial lung disease (ILD), Pulmonary embolism, Pulmonary hypertension, Cystic fibrosis, Coronary heart disease, Cardiomyopathy

[2]Specialist includes infectious disease specialist, pulmonologist, emergency physician, geriatrician

[3]Non-professional caregiver includes partner/spouse, parent/guardian, child under 18 years, child over 18 years, other relative(s), friend(s)/neighbor(s), other non-professional caregiver(s).

†Caution low base size

Confidential - Not for Public Consumption or Distribution

Supplementary Table S7: Resource utilization, caregiving needs and productivity loss – by country

	France				Germany				Italy				Spain				United Kingdom			
	**A-RSV (n=25[†])**	**O-RSV (n=31[†])**	**P-RSV (n=83)**	**H-RSV (n=74)**	**A-RSV (n=92)**	**O-RSV (n=45)**	**P-RSV (n=142)**	**H-RSV (n=159)**	**A-RSV (n=52)**	**O-RSV (n=70)**	**P-RSV (n=179)**	**H-RSV (n=102)**	**A-RSV (n=54)**	**O-RSV (n=36)**	**P-RSV (n=134)**	**H-RSV (n=147)**	**A-RSV (n=14[†])**	**O-RSV (n=14[†])**	**P-RSV (n=50)**	**H-RSV (n=78)**
**Visited a PCP, n (%)**	24 (96.0%)	26 (83.9%)	71 (85.5%)	38 (51.4%)	65 (70.7%)	31 (68.9%)	109 (76.8%)	92 (57.9%)	31 (59.6%)	54 (77.1%)	145 (81.0%)	61 (59.8%)	53 (98.1%)	36 (100.0%)	126 (94.0%)	75 (51.0%)	14 (100.0%)	12 (85.7%)	47 (94.0%)	34 (43.6%)
**Visited a specialist[1], n (%)**	4 (16.0%)	11 (35.5%)	27 (32.5%)	70 (94.6%)	39 (42.4%)	18 (40.0%)	70 (49.3%)	155 (97.5%)	43 (82.7%)	51 (72.9%)	126 (70.4%)	101 (99.0%)	13 (24.1%)	13 (36.1%)	57 (42.5%)	137 (93.2%)	1 (7.1%)	4 (28.6%)	13 (26.0%)	74 (94.9%)
**Number of HCP visits per patient, mean (SD)**	4.5 (3.4)	3.6 (2.9)	4.5 (2.6)	3.8 (2.2)	2.6 (2.3)	3.9 (2.7)	4.7 (2.3)	5.1 (3.0)	3.0(2.4)	4.1 (3.0)	3.8 (2.4)	3.9 (3.4)	3.5 (3.0)	4.2 (2.7)	6.0(4.6)	5.9 (5.4)	2.6 (1.4)	2.9 (1.6)	3.4 (2.1)	2.7 (1.9)
PCP/ GP	4.2 (3.5)	3.0(3.1)	3.9 (3.0)	1.7 (2.5)	1.8 (2.2)	2.7 (3.2)	3.4 (2.9)	1.4 (2.4)	1.8 (2.2)	2.7 (2.9)	2.6 (2.1)	0.9 (1.9)	3.1 (2.9)	3.7 (2.6)	5.3 (4.6)	2.0 (3.9)	2.5 (1.4)	2.4 (1.8)	2.9 (2.0)	0.6 (1.2)
Infectious disease specialist	-	0.2 (0.7)	0.1 (0.5)	0.5 (1.0)	0.4 (1.3)	0.7 (1.8)	0.7 (1.6)	1.5 (2.8)	0.8 (1.1)	0.9 (1.3)	0.8 (1.3)	0.8 (1.3)	0.1 (0.6)	-	0.1 (0.6)	1.7 (4.0)	-	-	0.1 (0.6)	0.1 (0.5)
Pulmonologist	0.0(0.2)	0.3 (0.7)	0.5 (1.1)	1.3 (1.3)	0.5 (0.9)	0.8 (1.5)	1 0 (1.6)	2.1 (2.6)	0.6 (1.0)	0.5 (0.9)	0.7 (1.2)	1.8 (3.0)	0.0 (0.3)	0.1 (0.5)	0.3 (0.7)	1.3 (2.2)	-	0.4 (1.1)	0.3 (1.2)	1.4 (1.5)
Emergency physician	0.00 (0.2)	0.1 (0.3)	0.1 (0.3)	0.4 (0.5)	-	-	0.0 (0.1)	0.2 (0.4)	0.0 (0.0)	0.0 (0.2)	0.0 (0.2)	0.4 (0.8)	0.2 (0.4)	0.4 (0.6)	0.4 (0.7)	0.9 (0.8)	0.1 (0.3)	0.1 (0.4)	0.0 (0.2)	0.5 (0.5)
Geriatrician	0.3 (1.2)	-	0.0 (0.2)	0.0 (0.2)	-	-	-	0.4 (1.2)	0.1 (0.2)	0.1 (0.4)	0.1 (0.7)	0.4 (1.4)	-	-	0.0 (0.2)	0.1 (0.5)	-	-	0.1 (0.3)	0.2 (0.6)
**Number of tests (to diagnose or monitor) per patient, mean (SD)**	1.8 (2.1)	2.8 (1.8)	4.3 (6.3)	10.0 (10.0)	2.7 (1.8)	3.5 (4.7)	4.6 (4.4)	6.8 (4.9)	2.3 (2.0)	3.9 (4.1)	5.3 (6.7)	7.0 (6.0)	2.7 (2.1)	4.1 (2.9)	5.9 (6.2)	11.9 (8.1)	2.0 (1.2)	3.7 (2.7)	4.3 (4.2)	14.9 (23.6)
RT-PCR	0.6 (0.6)	1.0 (1.0)	1.1 (1.1)	1.5 (0.8)	1.2 (0.7)	1.4 (0.8)	1.9 (1.0)	1.7 (0.9)	0.6 (0.8)	0.7 (0.9)	0.9 (1.1)	1.1 (0.9)	0.4 (0.6)	0.5 (0.8)	0.7 (1.3)	1.2 (0.9)	0.6 (0.5)	0.9 (0.5)	0.9 (0.6)	0.9 (0.7)
Rapid antigen	0.6 (0.7)	1.0 (1.1)	1.0 (1.3)	0.4 (0.8)	0.2 (0.6)	0.4 (2.1)	0.7 (1.9)	0.9 (1.6)	0.9 (0.9)	1.2 (1.0)	1.3 (1.2)	0.9 (0.9)	0.9 (0.7)	1.0 (0.9)	1.2 (1.2)	0.9 (1.4)	0.7 (0.9)	0.3 (0.5)	0.4 (0.6)	0.7 (0.9)
Pulse oximetry	0.3 (1.2)	0.5 (1.3)	0.9 (1.7)	1.5 (1.8)	0.7 (1.1)	1.0 (2.5)	1.1 (2.0)	1.6 (2.5)	0.1 (0.6)	0.7 (1.2)	0.6 (1.4)	0.8 (2.1)	0.7 (1.6)	1.2 (1.3)	1.8 (3.3)	1.8 (3.3)	0.1 (0.4)	0.9 (1.2)	1.2 (1.6)	0.6 (1.3)
Chest X-ray	0.1 (0.3)	0.2 (0.6)	0.4 (0.6)	1.8 (1.2)	0.5 (0.6)	0.4 (0.7)	0.7 (0.8)	1.3 (1.1)	0.4 (0.6)	0.6 (0.7)	0.8 (0.9)	1.4 (1.2)	0.4 (0.6)	0.8 (0.7)	1.0 (1.2)	2.1 (1.1)	0.3 (0.7)	0.4 (0.6)	0.5 (0.7)	1.2 (1.2)
**Received prescribed medication (currently or previously), n (%)**	13 (52.0%)	14 (45.2%)	39 (47.6%)	44 (61.1%)	26 (28.3%)	10 (22.2%)	28 (19.9%)	102 (64.2%)	11 (21.2%)	30 (44.1%)	64 (36.8%)	58 (57.4%)	21 (38.9%)	15 (41.7%)	64 (49.2%)	78 (53.8%)	3 (21.4%)	2 (14.3%)	16 (32.7%)	21 (27.6%)
**Most recent medication prescribed, n (%)**	**n=13[†]**	**n=14[†]**	**n=39[†]**	**n=44[†]**	**n=26[†]**	**n=10[†]**	**n=28[†]**	**n=102**	**n=11[†]**	**n=30[†]**	**n=64**	**n=58**	**n=21[†]**	**n=15[†]**	**n=64**	**n=78**	**n=3[†]**	**n=2[†]**	**n=16[†]**	**n=21[†]**
Ribavirin	3 (23.1%)	10 (71.4%)	21 (53.8%)	30 (68.2%)	24 (92.3%)	8 (80%)	19 (67.9%)	85 (83.3%)	10 (90.9%)	20 (66.7%)	50 (78.1%)	35 (60.3%)	4 (19.0%)	6 (40.0%)	20 (31.3%)	49 (62.8%)	1 (33.3%)	1 (50.0%)	12 (75.0%)	9 (42.9%)
Antibiotic	2 (15.4%)	0 (0.0%)	2 (5.1%)	6 (13.6%)	0 (0.0%)	0 (0.0%)	0 (0.0%)	7 (6.9%)	1 (9.1%)	1 (3.3%)	1 (1.6%)	9 (15.5%)	0 (0.0%)	1 (6.7%)	1 (1.6%)	7 (9.0%)	1 (33.3%)	1 (50.0%)	0 (0.0%)	4 (19.0%)
Corticosteroid	0 (0.0%)	1 (7.1%)	0 (0.0%)	0 (0.0%)	0 (0.0%)	0 (0.0%)	0 (0.0%)	0 (0.0%)	0 (0.0%)	1 (3.3%)	0 (0.0%)	0 (0.0%)	1 (4.8%)	0 (0.0%)	0 (0.0%)	10 (12.8%)	0 (0.0%)	0 (0.0%)	0 (0.0%)	1 (4.8%)
Analgesic	0 (0.0%)	0 (0.0%)	0 (0.0%)	0 (0.0%)	0 (0.0%)	0 (0.0%)	0 (0.0%)	0 (0.0%)	0 (0.0%)	0 (0.0%)	1 (1.6%)	0 (0.0%)	3 (14.3%)	1 (6.7%)	5 (7.8%)	1 (1.3%)	0 (0.0%)	0 (0.0%)	0 (0.0%)	0 (0.0%)
Other	8 (61.5%)	3 (21.4%)	16 (41.0%)	9 (20.5%)	2 (7.7%)	2 (20.0%)	9 (32.1%)	10 (9.8%)	1 (9.1%)	8 (26.7%)	12 (18.8%)	16 (27.6%)	13 (61.9%)	7 (46.7%)	38 (59.4%)	12 (15.4%)	1 (33.3%)	0 (0.0%)	4 (25.0%)	7 (33.3%)
**Over-the-counter medication taken, n (%)**	**n=18[†]**	**n=24[†]**	**n=64**	**n=64**	**n=87**	**n=40[†]**	**n=121**	**n=116**	**n=47[†]**	**n=58**	**n=133**	**n=81**	**n=49[†]**	**n=32[†]**	**n=106**	**n=121**	**n=8[†]**	**n=10[†]**	**n=39[†]**	**n=60**
Paracetamol	16 (88.9%)	22 (91.7%)	58 (90.6%)	64 (100.0%)	15 (17.2%)	13 (32.5%)	27 (22.3%)	52 (44.8%)	25 (53.2%)	31 (53.4%)	68 (51.1%)	52 (64.2%)	43 (87.8%)	28 (87.5%)	98 (92.5%)	97 (80.2%)	7 (87.5%)	8 (80.0%)	32 (82.1%)	55 (91.7%)
Cough suppressants	12 (66.7%)	6 (25.0%)	17 (26.6%)	14 (21.9%)	57 (65.5%)	18 (45.0%)	49 (40.5%)	63 (54.3%)	16 (34.0%)	19 (32.8%)	59 (44.4%)	27 (33.3%)	19 (38.8%)	14 (43.8%)	54 (50.9%)	44 (36.4%)	2 (25.0%)	4 (40.0%)	14 (35.9%)	13 (21.7%)

Ibuprofen	2 (11.1%)	3 (12.5%)	9 (14.1%)	3 (4.7%)	72 (82.8%)	25 (62.5%)	89 (73.6%)	64 (55.2%)	20 (42.6%)	24 (41.4%)	66 (49.6%)	34 (42.0%)	11 (22.4%)	10 (31.3%)	28 (26.4%)	16 (13.2%)	3 (37.5%)	3 (30.0%)	12 (30.8%)	13 (21.7%)
Nasal decongestants	7 (38.9%)	7 (29.2%)	14 (21.9%)	16 (25.0%)	48 (55.2%)	8 (20.0%)	33 (27.3%)	47 (40.5%)	17 (36.2%)	18 (31.0%)	53 (39.8%)	12 (14.8%)	14 (28.6%)	6 (18.8%)	29 (27.4%)	26 (21.5%)	3 (37.5%)	4 (40.0%)	13 (33.3%)	14 (23.3%)
Other	1 (5.6%)	0 (0.0%)	0 (0.0%)	4 (6.3%)	4 (4.6%)	2 (5.0%)	6 (5.0%)	9 (7.8%)	2 (4.3%)	3 (5.2%)	7 (5.3%)	5 (6.2%)	2 (4.1%)	7 (21.9%)	11 (10.4%)	35 (28.9%)	0 (0.0%)	0 (0.0%)	2 (5.1%)	2 (3.3%)
**Received supportive care (currently), n (%)**	**n=25†**	**n=31†**	**n=82**	**n=72**	**n=91**	**n=38†**	**n=134**	**n=153**	**n=52**	**n=68**	**n=178**	**n=101**	**n=54**	**n=36†**	**n=133**	**n=145**	**n=13†**	**n=14†**	**n=48†**	**n=78**
	13 (52.0%)	10 (32.3%)	27 (32.9%)	27 (37.5%)	28 (30.8%)	15 (39.5%)	48 (35.8%)	69 (45.1%)	12 (23.1%)	23 (33.8%)	41 (23.0%)	36 (35.6%)	18 (33.3%)	11 (30.6%)	61 (45.9%)	89 (61.4%)	4 (30.8%)	4 (28.6%)	14 (29.2%)	19 (24.4%)
**Supportive care type**	**n=13**	**n=10**	**n=27**	**n=27**	**n=28**	**n=15**	**n=48**	**n=69**	**n=12**	**n=23**	**n=41**	**n=36**	**n=18**	**n=11**	**n=61**	**n=89**	**n=4**	**n=4**	**n=14**	**n=19**
Non-professional care	11 (84.6%)	10 (100.0%)	26 (96.3%)	26 (96.3%)	28 (100.0%)	15 (100.0%)	47 (97.9%)	68 (98.6%)	12 (100.0%)	22 (95.7%)	38 (92.7%)	35 (97.2%)	16 (88.9%)	11 (100.0%)	57 (93.4%)	84 (94.4%)	3 (75.0%)	4 (100.0%)	11 (78.6%)	15 (78.9%)
Professional caregiver(s)	2 (15.4%)	1 (10.0%)	3 (11.1%)	6 (22.2%)	0 (0.0%)	2 (13.3%)	3 (6.3%)	1 (1.4%)	0 (0.0%)	3 (13.0%)	3 (7.3%)	2 (5.6%)	2 (11.1%)	1 (9.1%)	8 (13.1%)	10 (11.2%)	1 (25.0%)	0 (0.0%)	3 (21.4%)	7 (36.8%)
**Total number of care hours per week per patient, mean (SD)**	**n=5†**	**n=8†**	**n=15†**	**n=11†**	**n=20†**	**n=9†**	**n=33†**	**n=37†**	**n=5†**	**n=15†**	**n=14†**	**n=7†**	**n=12†**	**n=5†**	**n=37†**	**n=49†**	**n=2†**	**n=2†**	**n=5†**	**n=3†**
Non-professional[3]	12 (11.0)	8.1 (6.5)	11 (12.5)	13.7 (9.7)	22.8 (9.2)	25.8 (12.9)	20.2 (11.2)	19.5 (10.8)	16.0 (14.6)	32 (34.4)	20.9 (17.9)	29.9 (16.1)	47.8 (51.8)	24.6 (17.1)	52.3 (53.2)	49.4 (43.3)	25.0 (7.1)	3.5 (0.7)	15.0 (10.3)	10.0 (8.7)
Professional	**n=1†**	**n=0**	**n=3†**	**n=3†**	**n=0**	**n=2†**	**n=1†**	**n=0**	**n=0**	**n=2†**	**n=2†**	**n=2†**	**n=1†**	**n=1†**	**n=7†**	**n=9†**	**n=1†**	**n=0**	**n=2†**	**n=5†**
	12.0 (-)	- (-)	16.0 (13.5)	7.7 (3.8)	- (-)	6.5 (0.7)	10.0 (-)	- (-)	- (-)	35.0 (7.1)	27.5 (24.8)	40.0 (42.4)	40.0 (-)	20.0 (-)	80.3 (57.1)	52.8 (53.6)	40.0 (-)	- (-)	17.5 (14.9)	15.6 (15.3)
**Hospitalization**	**n=0**	**n=0**	**n=0**	**n=74**	**n=0**	**n=0**	**n=0**	**n= 159**	**n=0**	**n=0**	**n=0**	**n=102**	**n=0**	**n=0**	**n=0**	**n=147**	**n=0**	**n=0**	**n=0**	**n=78**
**Discharged on same day as admission, n (%)**	-	-	-	49 (83.1%)	-	-	-	117 (94.4%)	-	-	-	66 (93.0%)	-	-	-	130 (94.9%)	-	-	-	55 (90.2%)
**Requiring one or more overnight stays, n (%)**	-	-	-	10 (16.9%)	-	-	-	7 (5.6%)	-	-	-	5 (7.0%)	-	-	-	7 (5.1%)	-	-	-	6 (9.8%)
Total number of nights spent in hospital, mean (SD)	-	-	-	10.9 (8.9)	-	-	-	5.9 (3.9)	-	-	-	8.4 (5.4)	-	-	-	7.2 (4.7)	-	-	-	-
Total number of nights spent in hospital (patients never admitted to ICU), mean (SD)	**n=0**	**n=0**	**n=0**	**n=44**	**n=0**	**n=0**	**n=0**	**n=80**	**n=0**	**n=0**	**n=0**	**n=60**	**n=0**	**n=0**	**n=0**	**n=111**	**n=0**	**n=0**	**n=0**	**n=54**
	-	-	-	10.2 (8.7)	-	-	-	4.8 (2.9)	-	-	-	8.1 (5.2)	-	-	-	6.4 (3.9)	-	-	-	5.1 (4.2)
Total number of nights spent in hospital (patients admitted to ICU), mean (SD)	**n=0**	**n=0**	**n=0**	**n=5†**	**n=0**	**n=0**	**n=0**	**n=37†**	**n=0**	**n=0**	**n=0**	**n=6†**	**n=0**	**n=0**	**n=0**	**n=18†**	**n=0**	**n=0**	**n=0**	**n=1†**
	-	-	-	17.0 (8.5)	-	-)	-	8.3 (4.6)	-	-	-	11.8 (7.1)	-	-	-	11.5 (5.9)	-	-	-	8.0 (-)
**Admitted to ICU, n (%)**	**n=0**	**n=0**	**n=0**	**n=61**	**n=0**	**n=0**	**n=0**	**n=126**	**n=0**	**n=0**	**n=0**	**n=83**	**n=0**	**n=0**	**n=0**	**n=140**	**n=0**	**n=0**	**n=0**	**n=65**
	-	-	-	6 (9.8%)	-	-	-	37 (29.4%)	-	-	-	6 (7.2%)	-	-	-	19 (13.6%)	-	-	-	1 (1.5%)
Nights spent in ICU, mean (SD) (of those admitted to ICU)	-	-	-	4.2 (1.5)	-	-	-	2.7 (1.1)	-	-	-	3.2 (2.2)	-	-	-	4.1 (3.0)	-	-	-	5.0 (0.0)
**Received procedures for most recent infection while hospitalized, n (%)**	-	-	-	63 (85.1%)	-	-	-	104 (62.9%)	-	-	-	75 (73.5%)				127 (86.4%)				36 (46.2%)

**Received procedures for most recent infection while hospitalized, n (%)**	**n=0**	**n=0**	**n=0**	**n=63**	**n=0**	**n=0**	**n=0**	**n=104†**	**n=0**	**n=0**	**n=0**	**n=75**	**n=0**	**n=0**	**n=0**	**n=127**	**n=0**	**n=0**	**n=0**	**n=36†**
Receiving oxygen/IV fluids	-	-	-	62 (98.4%)	-	-	-	109 (95.6%)	-	-	-	71 (94.7%)	-	-	-	121 (95.3%)	-	-	-	36 (100.0%)
Nasal bulb suctioning	-	-	-	10 (15.9%)	-	-	-	18 (15.8%)	-	-	-	10 (13.3%)	-	-	-	16 (12.6%)	-	-	-	0 (0.0%)
Mechanical ventilation	-	-	-	2 (3.2%)	-	-	-	9 (7.9%)	-	-	-	6 (8%)	-	-	-	27 (21.3%)	-	-	-	0 (0.0%)
Receiving feeding tubes	-	-	-	1 (1.6%)	-	-	-	2 (1.8%)	-	-	-	9 (12%)	-	-	-	8 (6.3%)	-	-	-	0 (0.0%)
Intubation	-	-	-	1 (1.6%)	-	-	-	1 (0.9%)	-	-	-	4 (5.3%)	-	-	-	7 (5.5%)	-	-	-	0 (0.0%)
**WPAI: percentage overall work impairment, mean (SD)**	**n=0**	**n=1†**	**n=2†**	**n=0**	**n=3†**	**n=2†**	**n=3†**	**n=3†**	**n=1†**	**n=0**	**n=2†**	**n=0**	**n=4†**	**n=3†**	**n=2†**	**n=2†**	**n=0**	**n=0**	**n=1†**	**n=0**
	-	80.4 (-)	45.0 (21.2)	-	43.3 (30.6)	61.3 (0.3)	44.8 (44.0)	46.7 (25.2)	42.9 (-)	-	69.0 (7.5)	-	53.2 (28.4)	36.6 (38.0)	56.6 (37.6)	63.3 (33.0)	-	-	96.5 (-)	-
**WPAI: percentage overall activity impairment, mean (SD)**	**n=10†**	**n=8†**	**n=12†**	**n=10†**	**n=59**	**n=22†**	**n=74**	**n=78**	**n=9†**	**n=5†**	**n=13†**	**n=7†**	**n=31†**	**n=7†**	**n=21†**	**n=30†**	**n=1†**	**n=1†**	**n=3†**	**n=4†**
	54.0 (31.3)	41.3 (23.6)	60.8 (25.4)	60.0 (26.7)	51.7 (20.7)	57.7 (26.0)	39.7 (21.7)	58.1 (23.4)	46.7 (24.0)	34.0 (15.2)	47.7 (26.2)	51.4 (21.9)	47.4 (23.4)	30.0 (29.4)	58.1 (24.2)	59.0 (27.0)	60.0 (-)	60.0 (-)	70.0 (17.3)	52.5 (42.7)
**Missed work due to RSV, n (%)**	0 (0.0%)	1 (100.0%)	0 (0.0%)	0 (0.0%)	4 (66.7%)	3 (100.0%)	3 (60.0%)	11 (78.6%)	2 (100.0%)	0 (0.0%)	2 (100.0%)	1 (100.0%)	2 (50.0%)	2 (66.7%)	2 (66.7%)	2 (66.7%)	0 (0.0%)	0 (0.0%)	1 (100.0%)	1 (100.0%)
Missed hours of paid work due to RSV in the past seven days, mean (SD)	- (-)	24.0 (-)	- (-)	- (-)	18.0 (17.9)	17.3 (12.1)	11.2 (13.3)	29.8 (21.4)	24.0 (17.0)	- (-)	4.5 (2.1)	36.0 (-)	7.0 (11.5)	11.3 (12.1)	14.3 (22.3)	17.0 (15.4)	- (-)	- (-)	30.0 (-)	32.0 (-)
**Missed unpaid activity due to RSV, n (%)**	**n=10†**	**n=8†**	**n=13†**	**n=8†**	**n=58**	**n=21†**	**n=73**	**n=75**	**n=6†**	**n=3†**	**n=10†**	**n=5†**	**n=26†**	**n=8†**	**n=17†**	**n=27†**	**n=1†**	**n=0**	**n=1†**	**n=3†**
	2 (20.0%)	3 (37.5%)	4 (30.8%)	2 (25.0%)	7 (12.1%)	0 (0.0%)	6 (8.2%)	14 (18.7%)	0 (0.0%)	0 (0.0%)	1 (10.0%)	2 (40.0%)	5 (19.2%)	3 (37.5%)	3 (17.6%)	15 (55.6%)	0 (0.0%)	-	0 (0.0%)	0 (0.0%)
	**n=2†**	**n=3†**	**n=4†**	**n=2†**	**n=7†**	**n=0†**	**n=6†**	**n=14†**	**n=0**	**n=0**	**n=1†**	**n=2†**	**n=5†**	**n=3†**	**n=3†**	**n=15†**	**n=0**	**n=0**	**n=0**	**n=0**
**Hours of unpaid activity lost per week, mean (SD)**	5.5 (5.5)	8.0 (4.9)	4.3 (2.9)	4.5 (2.5)	5.6 (3.9)	-	5.0 (3.8)	5.3 (6.1)	-	-	15.0 (0.0)	5.0 (5.0)	4.4 (2.6)	8.0 (11.3)	20.0 (14.1)	7.6 (8.0)	-	-	-	-
Supporting or caring for a family member	**n=0**	**n=1†**	**n=0**	**n=0**	**n=2†**	**n=0**	**n=2†**	**n=3†**	**n=0**	**n=0**	**n=1†**	**n=1†**	**n=3†**	**n=1†**	**n=3†**	**n=2†**	**n=0**	**n=0**	**n=0**	**n=0**
	-	2.0 (-)	-	-	7.0 (1.4)	-	8.0 (2.8)	10.3 (8.5)	-	-	15.0 (-)	10.0 (-)	4.67 (3.2)	0.0 (-)	20.0 (17.3)	9.5 (7.8)	-	-	-	-
Supporting or caring for a friend	**n=0**	**n=1†**	**n=0**	**n=0**	**n=0**	**n=0**	**n=0**	**n=0**	**n=0**	**n=0**	**n=0**	**n=0**	**n=0**	**n=1†**	**n=0**	**n=0**	**n=0**	**n=0**	**n=0**	**n=0**
	-	8.0 (-)	-	-	-	-	-	-	-	-	-	-	-	0.0 (-)	-	-	-	-	-	-
Assisting in household activities	**n=1†**	**n=0**	**n=2†**	**n=2†**	**n=3†**	**n=0**	**n=4†**	**n=5†**	**n=0**	**n=0**	**n=0**	**n=0**	**n=†2**	**n=2†**	**n=0**	**n=10†**	**n=0**	**n=0**	**n=0**	**n=0**
	7.0 (-)	-	5.0 (2.8)	4.5 (3.5)	6.3 (3.2)	-	3.0 (2.0)	6.6 (4.8)	-	-	-	-	4.0 (2.8)	12.0 (17.0)	-	9.2 (5.7)	-	-	-	-
Voluntary work	**n=1†**	**n=0**	**n=0**	**n=0**	**n=1†**	**n=0**	**n=1†**	**n=3†**	**n=0**	**n=0**	**n=0**	**n=0**	**n=0**	**n=1†**	**n=0**	**n=0**	**n=0**	**n=0**	**n=0**	**n=0**
	4.0 (-)	-	-	-	6.0 (-)	-	2.0 (-)	3.3 (1.2)	-	-	-	-	-	0.0 (-)	-	-	-	-	-	-
Other	**n=0**	**n=1†**	**n=1†**	**n=0**	**n=0**	**n=0**	**n=0**	**n=0**	**n=0**	**n=0**	**n=0**	**n=0**	**n=0**	**n=1†**	**n=0**	**n=1†**	**n=0**	**n=0**	**n=0**	**n=0**
	-	14.0 (-)	7.0 (-)	-	-	-	-	-	-	-	-	-	-	0.0 (-)	-	3.0 (-)	-	-	-	-

For HCP visits answers of "Don't know" were imputed as the mean value for other patients.

Abbreviations: PCP – primary care physician, RF – risk factors, HCP – health care provider, SD – standard deviation, RT-PCR – reverse-transcription polymerase chain reaction, WPAI – work productivity and impairment scale

[1]Specialist includes infectious disease specialist, pulmonologist, emergency physician, geriatrician

[2]Non-professional caregiver includes partner/spouse, parent/guardian, child under 18 years, child over 18 years, other relative(s), friend(s)/neighbor(s), other non-professional caregiver(s).

†Caution low base size

Confidential - Not for Public Consumption or Distribution

Supplementary Table 8: Average cost associated with RSV per person in Euros (€) split by age and risk factors for severe RSV

A-RSV								
	60+ years		60-74 years			75+ Years		
**Direct costs**	**With RF (n=147)**	**Without RF (n=90)**	**Total (n=188)**	**With RF (n=109)**	**Without RF (n=79)**	**Total (n=49)**	**With RF (n=38†)**	**Without RF (n=11†)**
**Total direct cost, mean (SD)**	349 (238)	273 (215)	308 (219)	330 (212)	278 (225)	367 (276)	403 (299)	240 (112)
HCP visits, mean (SD)	109 (92)	94 (68)	92 (66)	93 (69)	90 (62)	144 (124)	152 (130)	116 (102)
Testing, mean (SD)	139 (82)	105 (71)	128 (82)	145 (85)	105 (72)	119 (103)	123 (74)	104 (65)
Medication, mean (SD)	101(173)	75 (22)	88(156)	92 (159)	82 (22)	103 (189)	128 (209)	20 (19)
**Indirect costs**								
**Total supportive care cost, mean (SD)**	192 (528)	56 (196)	94 (239)	126 (271)	51 (178)	317 (823)	382 (913)	93 (308)
**Total patient paid productivity loss, mean (SD)**	**n=65**	**n=38†**	**n=82**	**n=49**	**n=33†**	**n=21†**	**n=16†**	**n=5†**
	41 (170)	70 (250)	65 (225)	55 (194)	81 (266)	0 (0)	0 (0)	0 (0)
**Total patient unpaid productivity loss, mean (SD)**	**n=60†**	**n=40†**	**n=82†**	**n=46†**	**n=36†**	**n=18†**	**n=14†**	**n=4†**
	1 (4)	1 (5)	1 (5)	1 (4)	1 (6)	0 (0)	0 (0)	0 (0)
**O-RSV**								
	60+ years		60-74 years			75+ years		
	**With RF (n=131)**	**Without RF (n=65)**	**Total (n=144)**	**With RF (n=84)**	**Without RF (n=60)**	**Total (n=52)**	**With RF (n=47)**	**Without RF (n=5†)**
**Direct costs**								
**Total healthcare cost, mean (SD)**	541 (415)	501 (442)	459 (358)	452 (317)	468 (411)	721 (524)	701 (514)	903 (645)
HCP visits, mean (SD)	142 (91)	106 (68)	115 (72)	123 (74)	104 (69)	173 (104)	177 (108)	135 (60)
Testing, mean (SD)	165 (102)	128 (68)	138 (72)	144 (73)	131 (69)	192 (131)	202 (133)	97 (51)
Medication, mean (SD)	234 (373)	267 (424)	206 (345)	185 (302)	234 (399)	356 (480)	322 (466)	671 (552)
**Indirect costs**								
**Total supportive care cost, mean (SD)**	217 (685)	49 (206)	58 (200)	80 (232)	28 (139)	447 (1,024)	461 (1,064)	307 (554)
**Total patient paid productivity loss, mean (SD)**	**n=30†**	**n=12†**	**n=33†**	**n=22†**	**n=11†**	**n=8†**	**n=7†**	**n=1†**
	0 (0)	1,431 (1,877)	520 (1,303)	0 (0)	1,561(1,91 1)	0 (0)	0 (0)	0 (0)
**Total patient unpaid productivity loss, mean (SD)**	**n=28†**	**n=11†**	**n=31†**	**n=21†**	**n=10†**	**n=8†**	**n=7†**	**n=1†**
	0 (0)	0 (0)	0 (0)	0 (0)	0 (0)	0 (0)	0 (0)	0 (0)
**P-RSV**								
	60+ years		60-74 years			75+ years		
**Direct costs**	**With RF (n=418)**	**Without RF (n=170)**	**Total (n=401)**	**With RF (n=276)**	**Without RF (n=125)**	**Total (n=187)**	**With RF (n=142)**	**Without RF (n=45)**
**Total healthcare cost, mean (SD)**	934 (958)	753 (958)	843 (901)	876 (838)	770 (1,027)	964 (1,075)	1,046 (1,151)	704 (742)
HCP visits, mean (SD)	169 (103)	142 (83)	148 (88)	156 (94)	130 (70)	189 (113)	193 (115)	175 (106)
Testing, mean (SD)	205 (150)	163 (110)	199 (138)	213 (145)	168 (114)	180 (147)	191 (159)	147 (97)
Medication, mean (SD)	560 (900)	449 (905)	496 (842)	507 (781)	472 (966)	595 (1,019)	662 (1,091)	383 (715)
**Indirect costs**								
**Total supportive care cost, mean (SD)**	217 (718)	118 (492)	113 (496)	127 (529)	82 (417)	353 (901)	395 (964)	221 (654)
**Total patient paid productivity loss, mean (SD)**	**n=78**	**n=38†**	**n=85**	**n=60**	**n=25†**	**n=31†**	**n=18†**	**n=13†**
	1,123 (3,516)	128 (787)	1,088 (3,404)	1,460 (3,954)	194 (970)	0 (0)	0 (0)	0 (0)
**Total patient unpaid productivity loss, mean (SD)**	**n=79**	**n=33†**	**n=86**	**n=62**	**n=24†**	**n=26†**	**n=17†**	**n=9†**
	0 (1)	0 (1)	0 (1)	0 (1)	0 (1)	0 (0)	0 (0)	0 (0)
**H-RSV**								
	60+ years		60-74 years			75+ years		

Direct costs	With RF (n=481)	Without RF (n=79)	Total (n=369)	With RF (n=310)	Without RF (n=59)	Total (n=191)	With RF (n=171)	Without RF (n=20†)
**Total direct cost, mean (SD)**	7,167 (5,678)	5,276 (4,354)	6,355 (4,988)	6,648 (5,165)	4,815 (3,586)	7,954 (6,373)	8,108 (6,415)	6,637 (5,996)
HCP visits, mean (SD)	197 (136)	187 (175)	190 (124)	191 (121)	183 (143)	206 (170)	207 (159)	201 (250)
Testing, mean (SD)	256 (129)	191 (99)	252 (133)	264 (135)	187 (99)	238 (115)	242 (116)	204 (99)
Medication, mean (SD)	526 (891)	446 (790)	503 (844)	532 (867)	356 (699)	537 (940)	517 (936)	712 (987)
Hospitalization, mean (SD)	5,969 (5,384)	4,164 (4,137)	5,221 (4,702)	5,497 (4,872)	3,771 (3,361)	6,669 (6,100)	6,826 (6,129)	5,324 (5,820)
Procedures, mean (SD)	249 (641)	307 (1,137)	218 (559)	192 (136)	342 (1,318)	330 (976)	345 (1,032)	206 (127)
**Indirect costs**								
**Total supportive care cost, mean (SD)**	210 (604)	76 (255)	148 (425)	167 (455)	48 (188)	274 (771)	287 (803)	158 (388)
**Total patient paid productivity loss, mean (SD)**	**n=93**	**n=19†**	**n=83**	**n=69**	**n=14†**	**n=29†**	**n=24†**	**n=5†**
	203 (1,003)	80 (350)	245 (1,069)	273 (1,158)	109 (408)	0 (0)	0 (0)	0 (0)
**Total patient unpaid productivity loss, mean (SD)**	**n=96**	**n=22†**	**n=93**	**n=76**	**n=17†**	**n=25†**	**n=20†**	**n=5†**
	3 (14)	4 (8)	3 (14)	3 (14)	5 (9)	3 (10)	3 (11)	0 (2)

Abbreviations: A-RSV - acute respiratory syncytial virus, O-RSV – ongoing RSV, P-RSV – post-acute RSV, H-RSV - hospitalized RSV, RF – risk factors for severe RSV, HCP – healthcare professionals
*Total cost per patient excluding patient-reported productivity loss.
†Caution low base size

Supplementary Table S9: Average cost associated with RSV per person in Euros (€) split by country and patient type

	France (n=213)	Germany (n=438)	Italy (n=403)	Spain (n=371)	United Kingdom (n=156)
**A-RSV**					
**Direct costs**	**n=25†**	**n=92**	**n=52**	**n=54**	**n=14†**
**Total direct cost, mean (SD)**	269 (214)	478 (388)	243 (178)	334 (229)	208 (94)
HCP visits, mean (SD)	137 (103)	60 (51)	74 (52)	184 (171)	82 (43)
Testing, mean (SD)	78 (46)	179 (93)	96 (92)	116 (71)	113 (70)
Medication, mean (SD)	54 (162)	240 (342)	72 (104)	34 (92)	13 (18)
**Indirect costs**					
**Total supportive care cost, mean (SD)**	123 (313)	126 (264)	22 (88)	190 (523)	187 (416)
**Total patient paid productivity loss, mean (SD)**	**n=9†**	**n=55**	**n=8†**	**n=30†**	**n=1†**
	0 (0)	43 (210)	58 (163)	79 (228)	0 (0)
**Total patient unpaid productivity loss, mean (SD)**	**n=9†**	**n=58**	**n=6†**	**n=26†**	**n=1†**
	0 (0)	1 (6)	0 (0)	0 (0)	0 (0)
**O-RSV**					
**Direct costs**	**n=31†**	**n=45†**	**n=70**	**n=36†**	**n=14†**
**Total direct cost, mean (SD)**	593 (561)	585 (555)	419 (287)	585 (475)	288 (112)
HCP visits, mean (SD)	107 (85)	91 (63)	97 (64)	223 (145)	91 (51)
Testing, mean (SD)	133 (79)	197 (116)	117 (105)	163 (89)	137 (74)
Medication, mean (SD)	353 (510)	297 (538)	205 (253)	199 (403)	61 (69)
**Indirect costs**					
**Total supportive care cost, mean (SD)**	99 (226)	137 (300)	119 (322)	178 (693)	12 (31)
**Total patient paid productivity loss, mean (SD)**	**n=8†**	**n=21†**	**n=5†**	**n=8†**	**n=0**
	529 (1,496)	420 (1,326)	0 (0)	603 (1,233)	-
	**n=8†**	**n=21†**	**n=3†**	**n=7†**	**n=0**

**Total patient unpaid productivity loss, mean (SD)**	0 (0)	0 (0)	0 (0)	0 (0)	-
**P-RSV**					
**Direct costs**	**n=83**	**n=142**	**n=179**	**n=134**	**n=50**
**Total direct cost, mean (SD)**	829 (891)	986 (1,133)	731 (692)	931 (1,064)	730 (801)
HCP visits, mean (SD)	139 (81)	114 (58)	101 (54)	326 (240)	127 (72)
Testing, mean (SD)	154 (132)	272 (155)	483 (663)	215 (188)	167 (101)
Medication, mean (SD)	535 (804)	600 (1,109)	147 (130)	391 (910)	435 (778)
**Indirect costs**					
**Total supportive care received cost, mean (SD)**	105 (324)	121 (257)	45 (227)	359 (802)	61 (189)
**Total patient paid productivity loss, mean (SD)**	**n=11†**	**n=72**	**n=11†**	**n=19†**	**n=3†**
	1,268 (3,006)	395 (2,467)	1,577 (3,521)	770 (2,577)	5,201 (9,008)
**Total patient unpaid productivity loss, mean (SD)**	**n=11†**	**n=73**	**n=10†**	**n=17†**	**n=1†**
	0 (1)	0 (0)	0 (0)	0 (0)	0 (0)
**H-RSV**					
**Direct costs**	**n=74**	**n=159**	**n=102**	**n=147**	**n=78**
**Total direct cost, mean (SD)**	6,127 (6,159)	8,279 (5,467)	6,506 (6,127)	6,035 (5,392)	4,600 (4,549)
HCP visits, mean (SD)	123 (73.)	176 (98)	119 (87)	428 (369)	119 (91)
Testing, mean (SD)	236 (107)	259 (124)	181 (119)	336 (176)	210 (102)
Medication, mean (SD)	805 (1,016)	822 (1,396)	374 (520)	519 (882)	68 (136)
Hospitalization, mean (SD)	4,963 (5,817)	7,021 (5,328)	5,789 (6,087)	4,476 (4,975)	3,934 (4,441)
Procedures, mean (SD)	0 (0)	0 (1)	45 (436)	314 (952)	289 (303)
**Indirect costs**					
**Total supportive care cost, mean (SD)**	110 (305)	115 (249)	55 (202)	328 (654)	45 (207)
**Total patient paid productivity loss, mean (SD)**	**n=8†**	**n=67**	**n=6†**	**n=29†**	**n=2†**
	0 (0)	209 (1,067)	0 (0)	267 (1,068)	0 (0)
**Total patient unpaid productivity loss, mean (SD)**	**n=8†**	**n=75**	**n=5†**	**n=27†**	**n=3†**
	0 (0)	2 (8)	0 (0)	6 (24)	0 (0)

Abbreviations: A-RSV - acute respiratory syncytial virus, O-RSV – ongoing RSV, P-RSV – post-acute RSV, H-RSV - hospitalized RSV, RF – risk factors for severe RSV, HCP – healthcare professionals
†Caution low base size

References

1. Delaporte A. Le coût de la main-d'œuvre en France en 2020 : 38,7 euros par heure travaillée - Insee Focus - 283. www.insee.fr. Published January 4, 2023. Accessed May 30, 2025. https://www.insee.fr/fr/statistiques/6685426
2. Detasis Statistisches Bundesamt. Average hourly wage of persons employed. destatis.de. Published 2024. Accessed May 30, 2025. https://www.destatis.de/EN/Themes/Labour/Labour-Market/Quality-Employment/Dimension2/2_5_HourlyEarnings.html
3. Adonopoulos J. Stipendio medio Italia 2024: trend e statistiche. Forbes Advisor Italia. Published January 26, 2024. Accessed May 30, 2025. https://www.forbes.com/advisor/it/business/stipendio-medio-italia/
4. Instituto Nacional de Estadística. Salario anual medio, mediano y modal. Salario por hora. Brecha salarial de género (no ajustada) en salarios por hora. Published 2021. Accessed May 30, 2025. https://www.ine.es/jaxiT3/Datos.htm#_tabs-tabla
5. White N. Employee earnings in the UK - Office for National Statistics. www.ons.gov.uk. Published November 1, 2023. Accessed May 30, 2025.
6. Die bundesregierung. Mindestlohn für Pflegekräfte steigt | Bundesregierung. Die Bundesregierung informiert | Startseite. Published April 23, 2024. Accessed May 30, 2025. https://www.bundesregierung.de/breg-de/aktuelles/mindestlohn-altenpflege-steigt-2216632
7. D. Integlia, L. Di Censi, F. Di Giuseppe. La dimensione economica del caregiving formale e informale nel paziente con Fibrillazione Atriale. Clinico Economics. 2017;12:89-102.

Confidential - Not for Public Consumption or Distribution

doi:https://clinicoeconomics.eu/la-dimensione-economica-del-caregiving-formale-e-informale-nel-paziente-con-fibrillazione-atriale-clinico-economics-vol12-2017-pag89-102/
8. Moreno JO, Guerrero RO. Los costes de los cuidados informales en España. Presupuesto y gasto público. 2009;(56):163-181. doi:https://www.ief.es/docs/destacados/publicaciones/revistas/pgp/56_11.pdf
9. Chapter 11 page 122PSSRU. Unit Costs of Health and Social Care 2021: Unit Costs of Professionals. 2021. Accessed 18/04/2023. https://view.officeapps.live.com/op/view.aspx?src=https%3A%2F%2Fwww.pssru.ac.uk%2Fwp-content%2Fuploads%2F2021%2F12%2Funit-cost-of-health-and-social-care-staff-2020-21.xlsx&wdOrigin=BROWSELINK
10. Ameli.fr l'Assurance Maladie. Tarifs conventionnels des médecins généralistes et spécialistes. Ameli.fr. Published June 30, 2025. Accessed June 30, 2025. https://www.ameli.fr/medecin/exercice-liberal/facturation-remuneration/consultations-actes/tarifs/tarifs-conventionnels-medecins-generalistes-specialistes
11. Reimbursement.INFO. 04231 - Gespräch, Beratung und/oder Erörterung. Reimbursement.info. Published 2025. Accessed May 30, 2025. https://app.reimbursement.info/gops/-4231
12. Ministry of the Presidency, Justice and Local Administration of the Community of Madrid. Orden 1975/2023, de 29 de diciembre, de la Consejera de Sanidad, por la que se fijan los precios públicos por la prestación de los servicios y actividades de naturaleza sanitaria de la Comunidad de Madrid. Legislación de la Communidad de Madrid. Published December 29, 2023. Accessed May 30, 2025. https://gestiona.comunidad.madrid/wleg_pub/servlet/Servidor?opcion=VerHtml&nmnorma=13510
13. DEPARTAMENTO DE SALUD. SLT/30/2013, de 20 de febrero, por la que se aprueban los precios públicos del Servicio Catalán de la Salud. Diari Oicial de la Generalitat de Catalunya. Published February 26, 2013. Accessed October 1, 2024. https://www.ias.cat/adjunts/files/1286875(1).pdf
14. CONSEJERÍA DE SALUD Y CONSUMO. Orden de 24 de Mayo de 2024, Por La Que Se Establece El Importe de Los Servicios, Actividades Y Bienes de Naturaleza Sanitaria, Prestados En Centros Sanitarios Del Sistema Sanitario Público de Andalucía, Que Deben Ser Retribuidos Mediante Precios Públicos Por Los Terceros Obligados Legalmente al Pago. Boletín Oficial de la Junta de Andalucía; 2024:45904/1. Accessed October 1, 2024. https://www.sspa.juntadeandalucia.es/servicioandaluzdesalud/sites/default/files/sincfiles/wsas-media-sas_normativa_mediafile/2024/BOJA240605_importes.pdf
15. NHS England Digital. Unit cost of health and social care staff 2020-21. Published December 2021. Accessed May 30, 2025. https://view.officeapps.live.com/op/view.aspx?src=https%3A%2F%2Fwww.pssru.ac.uk%2Fwp-content%2Fuploads%2F2021%2F12%2Funit-cost-of-health-and-social-care-staff-2020-21.xlsx&wdOrigin=BROWSELINK
16. Baumgärtner W. Vertrag Zur Versorgung in Dem Fachgebiet Der Pneumologie in Baden-Württemberg Gemäß § 140a SGB v Zwischen AOK Baden-Württemberg ("AOK") Bosch BKK ("BKK"). AOK-FacharztProgramm / Bosch BKK-Facharztprogramm Pneumologie; 2021. Accessed 5, 2025. https://www.aok.de/gp/fileadmin/user_upload/Arzt_Praxis/Aerzte_Psychotherapeuten/AOK-FacharztProgramm_Baden-Wuerttemberg/bw_gesamtvertrag_pneumologie.pdf
17. Ministero della Salute. Salute.gov.it. Published 2025. Accessed May 30, 2025. https://www.salute.gov.it/portale/temi/p2_6.jsp?lingua=italiano&id=3662&area=programmazioneSanitariaLea&menu=vuoto
18. Reimbursement.INFO. 50512 - Grundpauschale ab 60. Lebensjahr. Reimbursement.info. Published 2025. Accessed May 30, 2025. https://app.reimbursement.info/gops/50512
19. Reimbursement.INFO. 01212 - Notfallpauschale II. Reimbursement.info. Published 2025. Accessed May 30, 2025. https://app.reimbursement.info/gops/01212
20. Reimbursement.INFO. 01210 - Notfallpauschale I. Reimbursement.info. Published 2025. Accessed May 30, 2025. https://app.reimbursement.info/gops/01210
21. Reimbursement.INFO. 03360 - Hausärztlich-geriatrisches Basisassessment. Reimbursement.info. Published 2025. Accessed May 30, 2025. https://app.reimbursement.info/gops/03360
22. Ameli.fr l'Assurance Maladie. Base des Médicaments et Informations Tarifaires. Ameli.fr. Published 2025. Accessed May 30, 2025.

Confidential - Not for Public Consumption or Distribution

http://www.codage.ext.cnamts.fr/codif/bdm_it//fiche/index_fic_medisoc.php?p_code_cip=3400941682803&p_site=AMELI
23. CGM. LAUER-TAXE®: for medicines, medical products & standard pharmacy goods. CGM Deutschland | Software für Arztpraxen, Apotheken & Kliniken. Published 2022. Accessed May 30, 2025. https://www.cgm.com/deu_de/produkte/apotheke/lauer-taxe-en.html
24. Vidal Vademecum. RIBAVIRINA NORMON 200 mg Comp. recub. con película - Prospecto. Vademecum.es. Published 2024. Accessed May 30, 2025. https://www.vademecum.es/espana/prospecto/37309/ribavirina-normon-200-mg-comprimidos-recubiertos-con-pelicula-efg
25. National Institute for Health and Care Excellence. Ribavirin Medicinal Forms. bnfc.nice.org.uk. Published 2025. Accessed April 30, 2025. https://bnfc.nice.org.uk/drugs/ribavirin/medicinal-forms/
26. Ministére Du Travail, De La Santé, Des Solidarités Et Des Familles. Base de Données Publique des Médicaments. Gouv.fr. Published December 11, 1993. Accessed May 30, 2025. https://base-donnees-publique.medicaments.gouv.fr/medicament/62365493/extrait
27. Festbetragsarzneimittel nach 35 SGB V, sortiert nach Festbatragrsgruppe und Wirkstoff. Published online January 15, 2025. Accessed April 30, 2025. https://www.bfarm.de/SharedDocs/Downloads/DE/Arzneimittel/Zulassung/amInformationen/Festbetraege/2025/festbetraege-20250115.pdf?__blob=publicationFile
28. AIFA. Lista Farmaci Equivalenti Nome Commerciale.; Published online April 14, 2025. Accessed May 30, 2025. https://www.aifa.gov.it/documents/20142/2833707/Lista_farmaci_equivalenti_Nome_Commerciale_14.04.2025.pdf.
29. Ministerio De Sanidad. Ministerio de Sanidad - Profesionales de la Salud - Nomenclator. Sanidad.gob.es. Published March 1, 2011. Accessed May 30, 2025. https://www.sanidad.gob.es/profesionales/nomenclator.do?metodo=verDetalle&prod=672066
30. National Institute for Health and Care Excellence. Dexamethasone Medicinal Forms. bnfc.nice.org.uk. Published 2025. Accessed April 30, 2025. https://bnf.nice.org.uk/drugs/dexamethasone/medicinal-forms/
31. Ministére Du Travail, De La Santé, Des Solidarités Et Des Familles. Base de Données Publique des Médicaments. Gouv.fr. Published November 16, 2009. Accessed May 30, 2025. https://base-donnees-publique.medicaments.gouv.fr/medicament/65819403/extrait
. 32. Ministerio De Sanidad. Ministerio de Sanidad - Profesionales de la Salud - Nomenclator. Sanidad.gob.es. Published June 1, 2013. Accessed May 30, 2025. https://www.sanidad.gob.es/profesionales/nomenclator.do?metodo=verDetalle&prod=694013
33. National Institute for Health and Care Excellence. Levofloxacin Medicinal Forms. bnfc.nice.org.uk. Published 2025. Accessed April 30, 2025. https://bnf.nice.org.uk/drugs/levofloxacin/medicinal-forms/#oral-tablet
34. Ministére Du Travail, De La Santé, Des Solidarités Et Des Familles. Base de Données Publique des Médicaments. Gouv.fr. Published November 16, 2009. Accessed May 30, 2025. https://base-donnees-publique.medicaments.gouv.fr/medicament/67436923/extrait
35. Ministerio De Sanidad. Ministerio de Sanidad - Profesionales de la Salud - Nomenclator. Sanidad.gob.es. Published January 1, 2008. Accessed May 30, 2025. https://www.sanidad.gob.es/profesionales/nomenclator.do?metodo=verDetalle&prod=656712
36.National Institute for Health and Care Excellence. Diclofenac potassium Medicinal Forms. bnfc.nice.org.uk. Published 2025. Accessed April 30, 2025. https://bnf.nice.org.uk/drugs/diclofenac-potassium/medicinal-forms/
37. MonCoinSanté. Ibuprofen Set 400 mg - Adults and children over 20 kg - 12 tablets. Pharmacie en ligne MonCoinSanté. Published 2025. Accessed July 11, 2025. https://moncoinsante.com/mcs/en/ibuprofen/16544-ibuprofen-set-400-mg-adults-and-children-over-20-kg-12-tablets-3400930179284.html
38. Apohealth. Ibuprofen axicur 400 mg akut Filmtabletten, 20 St FTA. apohealth - Gesundheit aus der Apotheke. Published 2024. Accessed July 14, 2025. https://www.apohealth.de/products/ibuprofen-axicur-400-mg-akut-filmtabletten-20-st-fta-18379483
39. Boletín Oficial del Estado. BOE-A-2024-20293 Orden SND/1074/2024, de 2 de octubre, por la que se procede a la actualización en 2024 del sistema de precios de referencia de medicamentos en el

Sistema Nacional de Salud. Www.boe.es. Published August 8, 2024. Accessed May 30, 2025. https://www.boe.es/eli/es/o/2024/10/02/snd1074
40. National Institute for Health and Care Excellence. Ibuprofen Medicinal Forms. bnfc.nice.org.uk. Published 2025. Accessed April 30, 2025. https://bnf.nice.org.uk/drugs/ibuprofen/medicinal-forms/
41. Euro-pharmas. DafalganCaps 500 mg - 16 Capsules - Upsa. Euro-pharmas. Published 2025. Accessed July 14, 2025. https://euro-pharmas.com/en/buy-paracetamol-online-pharmacy/1364-1518-dafalgancaps-500-mg-16-capsules-upsa.html#/120-capacity-16_caps
42. Apohealth. Paracetamol ADGC 500 mg Tabletten bei Schmerzen oder Fieber, 20 pcs. Tablets. apohealth - Gesundheit aus der Apotheke. Published 2024. Accessed July 14, 2025. https://www.apohealth.de/en/products/paracetamol-adgc-500-mg-tabletten-bei-schmerzen-oder-fieber-20-st-tabletten-17502496
43. National Institute for Health and Care Excellence. Paracetamol Medicinal Forms. bnfc.nice.org.uk. Published 2025. Accessed April 30, 2025. https://bnf.nice.org.uk/drugs/paracetamol/medicinal-forms/#oral-tablet
44. Euro-pharmas. Nasal spray with essential oils 20 ml - NATURACTIVE. Euro-pharmas. Published 2025. Accessed July 14, 2025. https://euro-pharmas.com/en/nasal-spray/1593-1809-nasal-spray-with-essential-oils-20-ml-naturactive.html#/45-capacity-20_ml
45. Shop Apotheke. Fragen und Antworten zu Nasenspray-ratiopharm Erwachsene - bei Schnupfen. Shop-apotheke.com. Published 2025. Accessed July 14, 2025. https://www.shop-apotheke.com/arzneimittel/999831/nasenspray-ratiopharm-erwachsene.htm?cstrackid=05830ce0-dea2-48fa-a852-13779306f331&campaign=affiliate/awin/213931/&expa=awin&subid=Teva&sv1=affiliate&sv_campaign_id=213931&awc=13808_1747993604_a7890878a655b20521236f43c423fa63
46. Farmacia Rubino. Deltarinol Nasal Spray 0.5% + 0.125% Ephedrine Hydrochloride Bottle 15 ml. www.farmaciarubino.net. Published 2025. Accessed July 14, 2025. https://farmaciarubino.net/en/over-the-counter-drugs/134-deltarinol-nasal-spray-05-0125-ephedrine-hydrochloride-bottle-15-ml.html
47. Vidal Vademecum Spain. RHINOVÍN 1 mg/ml Sol. nebul. - FICHA TÉCNICA. Vademecum.es. Published 2024. Accessed July 14, 2025. https://www.vademecum.es/espana/ficha-tecnica/17646/rhinovin-1-mg-ml-solucion-para-pulverizacion-nasal
48. National Institute for Health and Care Excellence. Pseudophedrine hydrochloride Medicinal Forms. bnfc.nice.org.uk. Published 2025. Accessed April 30, 2025. https://bnf.nice.org.uk/drugs/pseudoephedrine-hydrochloride/medicinal-forms/#oral-solution
49. The French Pharmacy. Broncho Stop - Cough Syrup 120ml. thefrenchpharmacy.co. Published 2025. Accessed July 14, 2025. https://thefrenchpharmacy.co/collections/colds-coughs-sore-throats-cough/products/broncho-stop-120ml
50. Arzneiprivat. WICK MediNait cold syrup for the night. Arzneiprivat.de. Published 2025. Accessed July 14, 2025. https://www.arzneiprivat.de/product/wick-medinait-cold-syrup-for-the-night.127316.html?language_code=en
51. Farmacia Igea. Teofarma Cardiazol Paracodina Orale Gocce 10 Ml 20 Mg/ml + 100 Mg/ml. farmaciaigea.com. Published 2025. Accessed July 14, 2025. https://farmaciaigea.com/cardiazol-paracodinaorale-gtt-10-ml-20-mg-ml-100-mg-ml
52. Farmacia Escofet. Bisolvon Antitusivo Compositum jarabe 200 ml. Farmacia Escofet. Published 2025. Accessed July 14, 2025. https://farmaciaescofet.com/en/medicines/cough/cough-suppressant/207-bisolvon-antitusivo-compositum-jarabe-200-ml.html
53. LloydsPharmacy. Night Nurse liquid. LloydsPharmacy. Published 2025. Accessed July 14, 2025. https://lloydspharmacy.com/products/night-nurse-liquid-160ml?variant=39727530934335
54. Cohen L, Génisson C, Savary RP. Rapport D´Information. Présidence du Sénat; 2017. Accessed May 30, 2025. https://www.senat.fr/rap/r16-685/r16-6851.pdf
55. Haas C, Larbig M, Schöpke T, et al. Gutachten Zur Ambulanten Notfallversorgung Im Krankenhaus - Fallkostenkalkulation Und Strukturanalyse Der Management Consult Kestermann GmbH (MCK) Erstellt in Kooperation Mit Der Deutsche Gesellschaft Interdisziplinäre Notfall-Und Akutmedizin E. V. (DGINA). Deutsche Gesellschaft interdisziplinäre Notfall - und Akutmedizin e.V.; 2015. Accessed July 30, 2025. https://www.dkgev.de/fileadmin/default/Mediapool/2_Themen/2.2_Finanzierung_und_Leistungskatal

Confidential - Not for Public Consumption or Distribution

oge/2.2.3._Ambulante_Verguetung/2.2.3.4._Ambulante_Notfallvehandlung_durch_Krankenhaeuser/2015-02-17_Gutachten_zur_ambulanten_Notfallversorgung_im_Krankenhaus_2015.pdf
56. Maccari C, La Torre G, Del Cimmuto A. Il dimensionamento delle centrali di emergenza sanitaria 118: dalla prassi alla teoria. researchgate.net. Published September 2009. Accessed May 30, 2025. https://www.researchgate.net/publication/236951377_Il_dimensionamento_delle_centrali_di_emergenza_sanitaria_118_dalla_prassi_alla_teoria?enrichId=rgreq-5506721f91fc5f4f89d08985b7b326f2-XXX&enrichSource=Y292ZXJQYWdlOzIzNjk1MTM3NztBUzo5OTkxMDA3MDMwODg3NUAxNDAwODMxODI1Mzky&el=1_x_2&_esc=publicationCoverPdf
57. Hodgson D, Wilkins N, van Leeuwen E, et al. Protecting infants against RSV disease: an impact and cost-effectiveness comparison of long-acting monoclonal antibodies and maternal vaccination. The Lancet Regional Health - Europe. 2024;38:100829. doi:https://doi.org/10.1016/j.lanepe.2023.100829
58. Nuttens C, Barbet V, Clélia Bignon-Favary, et al. Estimation of GP visits, hospitalizations and deaths attributable to RSV and influenza and costs associated with hospitalizations in older adults in France, 2010-2020. medRxiv (Cold Spring Harbor Laboratory). Published online April 30, 2025. doi:https://doi.org/10.1101/2025.04.29.25326541
59. Huebbe B, Mocek A, Manz KC, et al. Economic burden of respiratory syncytial virus in adults in Germany – a health claims analysis between 2015 and 2018. Journal of Medical Economics. 2024;27(1):1063-1075. doi:https://doi.org/10.1080/13696998.2024.2389676
60. Ministero dell'Economia e delle Finanze Commissione Tecnica per la Finanza Pubblica. Libro Verde Sulla Spesa Pubblica.; 2007. Accessed May 30, 2025. https://www.mef.gov.it/export/sites/MEF/ministero/commissioni/ctfp/documenti/Libro_verde_spesa_pubblica.pdf
61. Ministerio De Sanidad. Portal Estadístico - Área de Inteligencia de Gestión. Sanidad.gob.es. Published 2015. Accessed May 30, 2025. https://pestadistico.inteligenciadegestion.sanidad.gob.es/publicoSNS/S/rae-cmbd
62. NHS England. National Cost Collection: National Schedule of NHS Costs. Published 2022. Accessed July 25 2023. https://www.england.nhs.uk/costing-in-the-nhs/national-cost-collection/
63. NHS England Digital. Hospital Episode Statistics 2020-2021. Published 2022. Accessed July 25 2023. https://digital.nhs.uk/services/hospital-episode-statistics
64. Kaier K, Heister T, Motschall E, Hehn P, Bluhmki T, Wolkewitz M. Impact of mechanical ventilation on the daily costs of ICU care: a systematic review and meta regression. Epidemiol Infect. 2019;147:e314. Published 2019 Dec 5 2019. Accessed May 30 2025. doi:10.1017/S0950268819001900
65. Zwerwer LR, Kloka J, van der Pol S, et al. Mechanical ventilation as a major driver of COVID-19 hospitalization costs: a costing study in a German setting. Health Economics Review. 2024;14(1). doi:https://doi.org/10.1186/s13561-023-00476-1
66. Tan SS, Bakker J, Hoogendoorn ME, et al. Direct cost analysis of intensive care unit stay in four European countries: applying a standardized costing methodology. Value in Health: The Journal of the International Society for Pharmacoeconomics and Outcomes Research. 2012;15(1):81-86. doi:https://doi.org/10.1016/j.jval.2011.09.007
67. Gisbert R, Brosa M. Base de datos de costes sanitarios y ratios coste-efectividad españoles: eSalud [Internet]. Barcelona: Oblikue Consulting, S.L. 2007.
68. SYNLAB. Laborinformation / Mikrobiologie 18 Juni 2020 Respiratory-Syncytial-Virus.; 2023. Accessed May 30, 2025. https://www.labor-muenchen-zentrum.de/fileadmin/user_upload/Mikrobiologie/BAK-18-RSV.pdf
69. Scottish Government. Cost of processing a PCR test for Covid-19: FOI release. www.gov.scot. Published July 20, 2021. Accessed May 30, 2025. https://www.gov.scot/publications/foi-202100215784/
70. Reimbursement.INFO. 32788 - Nachweis von RSV. Reimbursement.info. Published 2025. Accessed May 30, 2025. https://app.reimbursement.info/gops/32788
71. Scandrett K, Colquitt J, Court R, et al. Evidence review for rapid tests to inform triage and antibiotic prescribing decisions: Suspected acute respiratory infection in over 16s: assessment at first presentation and initial management: Evidence review B. The BMJ. Published online October 2023:q339-q339. doi:https://doi.org/10.1136/bmj.q339

Confidential - Not for Public Consumption or Distribution

72. Reimbursement.INFO. 34220 - Aufnahmen des knöchernen Thorax. Reimbursement.info. Published 2025. Accessed May 30, 2025. https://app.reimbursement.info/gops/34220
73. NHS England Digital. 2 National schedule of NHS costs FY21-22 v3. Published December 2021. Accessed May 24, 2025. https://view.officeapps.live.com/op/view.aspx?src=https%3A%2F%2Fwww.england.nhs.uk%2Fwp-content%2Fuploads%2F2023%2F04%2F2_National_schedule_of_NHS_costs_FY21-22_v3.xlsx&wdOrigin=BROWSELINK
74. Abrechnungsstelle. GOÄ 602: Oxymetrische Untersuchung(en) (Bestimmung der prozentualen Sauerstoffsättigung im Blut) - gegebenenfalls einschließlich Bestimmung(en) nach Belastung - | abrechnungsstelle.com. abrechnungsstelle.com. Published June 14, 2023. Accessed May 30, 2025.
75. Timesco. Finger Tip Pulse Oximeters Available via NHS Supply Chain.; 2018. Accessed May 30, 2025. https://timesco.com/wp-content/uploads/2021/06/idco.pdf
76. Diego ONE. EBM 32720 Urinuntersuchung. Diego.one. Published 2025. Accessed May 30, 2025. https://diego.one/ebm/32720
77. National Institute for Health and Care Excellence. Overview | Routine preoperative tests for elective surgery | Guidance | NICE. Nice.org.uk. Published April 5, 2016. Accessed May 30, 2025. https://www.nice.org.uk/guidance/ng45
78. Diego ONE. EBM 32122 Mechanisierter vollständiger Blutstatus. Diego.one. Published 2025. Accessed May 30, 2025. https://diego.one/ebm/32122
79. Diego ONE. EBM 32721 Sputum-, Bronchialsekretuntersuchung. Diego.one. Published 2025. Accessed May 30, 2025. https://diego.one/ebm/32721
80. NHS Foundation Trust. Private Patient Tariff Issue 1 1 St.; 2023. Accessed May 30, 2025. https://www.dchft.nhs.uk/wp-content/uploads/2023/03/Private-Patient-Tariff-23-24-002.pdf
81. Reimbursement.INFO. 34220 - Aufnahmen des knöchernen Thorax. Reimbursement.info. Published 2025. Accessed May 30, 2025. https://app.reimbursement.info/gops/34220
82. Reimbursement.INFO. 01221 - Zuschlag Beatmung. Reimbursement.info. Published 2025. Accessed May 30, 2025. https://app.reimbursement.info/gops/01221
83. Merino M, Villoro R, Hidalgo-Vega Á, Carmona C. Social economic costs of COPD in Extremadura (Spain): an observational study. International Journal of Chronic Obstructive Pulmonary Disease. 2018;Volume 13(2018):2501-2514. doi:https://doi.org/10.2147/copd.s167357
84. Boletín Oficial del Estado. BOE-A-2021-21337 Resolución de 22 de diciembre de 2021, de la Mutualidad General de Funcionarios Civiles del Estado, por la que se publica el Concierto suscrito con entidades de seguro para el aseguramiento del acceso a la asistencia sanitaria en territorio nacional a los beneficiarios de la misma durante los años 2022, 2023 y 2024. Www.boe.es. Published December 24, 2021. Accessed May 30, 2025. https://www.boe.es/diario_boe/txt.php?id=BOE-A-2021-21337
85. Diego ONE. GOÄ 29977 Lobektomie/Lungensegmentresektion(en). Diego.one. Published 2025. Accessed May 30, 2025. https://diego.one/goä/2997
86. Crespo IR. Evaluación económica de la lobectomía pulmonar por cirugía videotoracoscópica en Aragón. Dialnet. Published 2016. Accessed June 5, 2025. https://dialnet.unirioja.es/servlet/tesis?codigo=203135
87. Osakidetza. TARIFAS PARA FACTURACIÓN de SERVICIOS SANITARIOS Y DOCENTES de OSAKIDETZA PARA EL AÑO 2025 Diciembre 2024.; 2024. Accessed May 30, 2025. https://www.osakidetza.euskadi.eus/contenidos/informacion/osk_servic_para_empresas/es_def/adjuntos/LIBRO-DE-TARIFAS-2025-CAS-FIRMADO.pdf
88. Jones K, Weatherly H, Chalkley A, et al. Unit Costs of Health and Social Care 2023 Manual. Personal Social Services; 2024. Accessed May 30, 2025. https://kar.kent.ac.uk/105685/1/The%20unit%20costs%20of%20health%20and%20social%20care_Final3.pdf
89. Paylab. Salaries in France - Paylab.com. Paylab - Salary survey, Compare salary, Salary data. Published 2025. Accessed May 15, 2025. https://france.paylab.com/salaires-dans-le-pays?lang=fr
90. Paylab. Salaries in Germany - Paylab.com. Paylab - Salary survey, Compare salary, Salary data. Published 2025. Accessed May 15, 2025. https://www.paylab.com/de/salaries-in-country?lang=en
91. Paylab. Salaries in Italy - Paylab.com. Paylab - Salary survey, Compare salary, Salary data. Published 2025. Accessed May 15, 2025. https://www.paylab.com/it/salaries-in-country?lang=en

Confidential - Not for Public Consumption or Distribution

92. Paylab. Salaries in Spain - Paylab.com. Paylab - Salary survey, Compare salary, Salary data. Published 2025. Accessed May 15, 2025. https://www.paylab.com/es/salaries-in-country?lang=en
93 Paylab. Salaries in United Kingdom - Paylab.com. Paylab - Salary survey, Compare salary, Salary data. Published 2025. Accessed May 15, 2025. https://gb.paylab.com/salaries-in-country?lang=en
